# Autonomous IL-36R signaling in neutrophils activates potent antitumor effector functions

**DOI:** 10.1172/JCI162088

**Published:** 2023-06-15

**Authors:** Sumedha Roy, Karen Fitzgerald, Almin Lalani, Chin-Wen Lai, Aeryon Kim, Jennie Kim, Peiqi Ou, Annie Mirsoian, Xian Liu, Ambika Ramrakhiani, Huiren Zhao, Hong Zhou, Haoda Xu, Hans Meisen, Chi-Ming Li, Bryan Vander Lugt, Steve Thibault, Christine E. Tinberg, Jason DeVoss, Jackson Egen, Lawren C. Wu, Rajkumar Noubade

**Affiliations:** 1 Oncology Therapeutic Area,; 2 Inflammation Therapeutic Area,; 3 Genome Analysis Unit, and; 4 Therapeutic Discovery, Amgen, South San Francisco, California, USA.

**Keywords:** Immunology, Oncology, Immunotherapy, Innate immunity, Neutrophils

## Abstract

While the rapid advancement of immunotherapies has revolutionized cancer treatment, only a small fraction of patients derive clinical benefit. Eradication of large, established tumors appears to depend on engaging and activating both innate and adaptive immune system components to mount a rigorous and comprehensive immune response. Identifying such agents is a high unmet medical need, because they are sparse in the therapeutic landscape of cancer treatment. Here, we report that IL-36 cytokine can engage both innate and adaptive immunity to remodel an immune-suppressive tumor microenvironment (TME) and mediate potent antitumor immune responses via signaling in host hematopoietic cells. Mechanistically, IL-36 signaling modulates neutrophils in a cell-intrinsic manner to greatly enhance not only their ability to directly kill tumor cells but also promote T and NK cell responses. Thus, while poor prognostic outcomes are typically associated with neutrophil enrichment in the TME, our results highlight the pleiotropic effects of IL-36 and its therapeutic potential to modify tumor-infiltrating neutrophils into potent effector cells and engage both the innate and adaptive immune system to achieve durable antitumor responses in solid tumors.

## Introduction

Immunotherapy, particularly checkpoint inhibitors such as anti-PD(L)1, has revolutionized treatment options for patients with cancer. However, responses are limited to only subsets of patients and indications (1–3). Intratumoral T cell presence of has been the most reliable prognostic factor for response to therapy. Consequently, cancer immunotherapy research has been focused on harnessing T cell–driven biology (4, 5). Recent technological advancements have resulted in better appreciation of the complexity and heterogeneity of the TME, pointing toward several immunosuppressive mechanisms to effect immunotherapy efficacy in solid tumors. Therefore, unsurprisingly, T cell–based therapies alone are unable to completely regress the most advanced solid tumors, suggesting that complementary approaches engaging both adaptive and innate immune cells are likely needed (6–8).

The IL-36 family of cytokines, members of the IL-1 superfamily, are powerful immunomodulatory agents, consisting of 3 activating ligands, IL-36α, IL-36β and IL-36γ, and an inhibitory ligand, IL-36RN. They are produced by multiple cell types, especially at the epithelial barrier interface, and are induced by cytokines, chemokines, and growth factors (9–11). They bind to a heterodimeric receptor consisting of IL-36 receptor (IL-36R, also known as IL-1Rrp2 or IL-1RL2) and its coreceptor IL-1 receptor accessory protein (IL-1RAcP) (11). IL-36R is expressed on many cell types, including epithelial, mesenchymal, and immune cells, such as T cells, γδT cells, NK cells, DCs, and macrophages (12, 13). Recently, IL-36R expression on neutrophils has also been demonstrated in mice and humans (14, 15). Ligand binding to IL-36R triggers MyD88-dependent signaling cascade and activation of the NF-κB and MAP kinase pathways (11, 16), resulting in induction of proinflammatory cytokines and chemokines (16). IL-36 has been demonstrated to be a key player in inflammation, autoimmunity, and fibrosis (9, 10, 17) due to its effects on both the innate and adaptive immune system, which induce, among other effects, proliferation; IFN-γ production; Th1 differentiation of CD4 T cells, γδT cells, and NK cells; and DC maturation and antigen presentation (13, 18). Recent publications have shown that the IL-36/IL-36R axis can impact antitumor immunity via its effects on the adaptive immune system (19–23). However, the role of innate immune cells, particularly tumor-associated neutrophils, in IL-36–mediated antitumor immunity remains poorly understood.

A subset of neutrophils, referred to as polymorphonuclear (PMN) myeloid-derived suppressor cells (MDSCs) (PMN-MDSCs), is generally associated with dampening T cell responses and poor patient prognosis (1, 3, 8). However, evidence is emerging in favor of antitumor functions of neutrophils, either via direct tumor cell killing, mostly through ROS, or indirectly through antibody-dependent cell cytotoxicity or their ability to recruit and activate T cells in the TME (24–27). In this regard, IL-36 is known to induce significant neutrophil influx in inflammation (14, 17) and cancer (20). IL-36 signaling amplifies neutrophil-mediated inflammatory reaction in the lung (14). Therefore, we focused on dissecting IL-36 signaling in neutrophils and its effect on antitumor immunity. We demonstrate that cell-autonomous IL-36 signaling in neutrophils turns them into potent antitumor effector cells. Our results highlight the plasticity of tumor-infiltrating neutrophils not only to acquire antitumor phenotypes but also to influence other antitumor effector cells. The ability of IL-36 signaling to trigger the remodeling of an otherwise immunosuppressive TME through direct effect on neutrophils emphasizes its potential to enhance efficacy of T cell–focused therapeutics, especially in indications of high unmet medical need where such immunosuppressive mechanisms are dominant.

## Results

### IL-36 elicits potent antitumor effects via host hematopoietic cells.

To evaluate antitumor effects of the IL-36/IL-36R signaling axis systematically, we examined an expansive set of preclinical tumor models representing different genetic backgrounds, diverse TMEs, and varied responses to cancer immunotherapies. These included B16F10 (melanoma, C57BL/6), Renca (renal adenocarcinoma, BALB/c), MC38 (colon carcinoma, C57BL/6), and CT26 (colon carcinoma, BALB/c) models. These tumor models also mimic broad categories of immune representation within human tumors, namely immunologically cold (B16F10 and Renca), immunologically warm but suppressive (MC38), and immunologically hot tumors (CT26). Because IL-36γ has previously been described as a potent proinflammatory agent, we used it as a representative of the IL-36 family of cytokines for our study (28).

**Figure 1:**
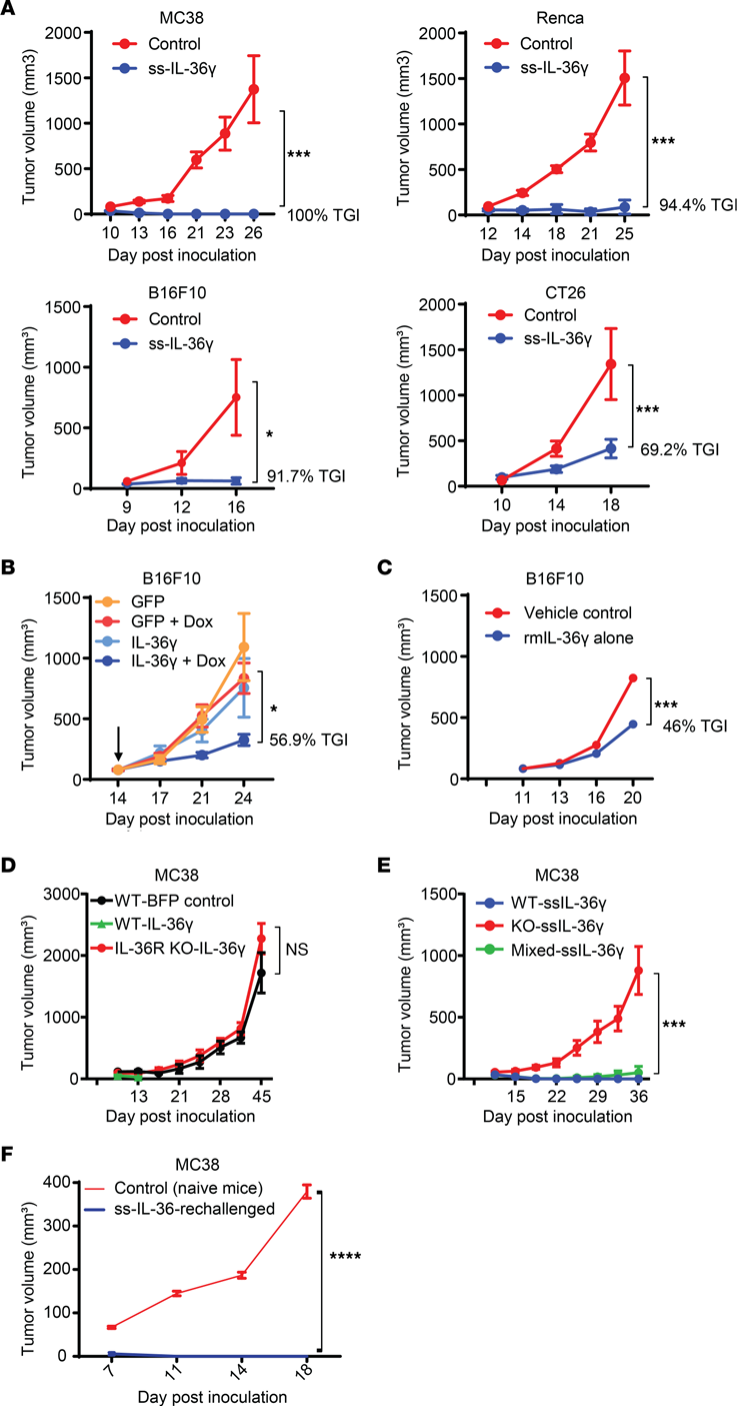
IL-36 exhibits potent antitumor immune response via effects on hematopoietic cells. (**A**) Tumor growth of control or IL-36γ–expressing MC38, B16F10, Renca, and CT26 syngeneic tumor models (*n* = 10 for each group in each model). (**B**) Tumor growth of control B16F10 cells or cells with doxycycline-inducible (Dox-inducible) expression of IL-36γ. The arrow indicates initiation of Dox treatment on day 14 (*n* = 10/ group). (**C**) Tumor growth of B16F10 tumor with intratumoral administration of either vehicle control or rmIL-36γ (*n* = 10/group). (**D**) Tumor growth of control or IL-36γ–expressing MC38 cells in WT C57BL/6 or IL-36R–KO mice (*n* = 5/group). (**E**) Tumor growth of control or IL-36γ–expressing MC38 cells in bone marrow chimera mice that were irradiated and transplanted with WT or KO or equal mix of the WT and KO hematopoietic cells (*n* = 10/group). (**F**) Tumor growth of MC38 cells in naive WT mice or in mice that rejected primary tumor expressing IL-36γ (60 days after being tumor free) (*n* = 10/group). Data are shown as mean ± SEM. Two-way ANOVA followed by Šidák’s multiple comparison test (2 groups) or Tukey’s multiple comparison test (>2 groups). *P < 0.05, ***P < 0.001, ****P < 0.0001. ss, signal sequence. Data are representative of 3 (**A**, **B**, and **D**) and 2 (**C**, **E**, and **F**) independent experiments.

To assess antitumor effects of the IL-36 pathway, we first established model systems by generating IL-36γ–expressing syngeneic tumor cell lines. Like other IL-1 family members, IL-36γ lacks a signal sequence (ss), and therefore we evaluated the effect of introducing an ectopic signal sequence to potentially enhance its secretion. We confirmed the bioactivity of secreted IL-36γ in supernatants of stably transfected tumor cell lines using a previously described (11) IL-36R reporter assay of Ba/F3 cells expressing mouse IL-36R and IL-8 promoter–driven NF-κB luciferase. As expected, cells expressing IL-36γ with an ectopic signal sequence secreted more IL-36γ than those without the signal sequence (Supplemental Figure 1A; supplemental material available online with this article; https://doi.org/10.1172/JCI162088DS1). We also confirmed that there was no impact of IL-36γ expression on tumor cell line growth in vitro (Supplemental Figure 1B). However, in vivo, enhanced IL-36γ expression led to significantly stunted growth of all 4 tumor cell lines compared with controls, including the cold and aggressive B16F10 and Renca tumor models (Figure 1A and Supplemental Figure 1C). Remarkably, MC38 and Renca tumors that grew for about a week initially were completely abolished in 100% of the mice (Figure 1A). Cell lines harboring IL-36γ without an ectopic signal sequence showed milder tumor control, consistent with lower IL-36γ protein expression compared with cells with ss-IL-36γ (IL-36γ with ectopic signal sequence), suggesting a dose-dependent effect of IL-36γ (Supplemental Figure 1C). Together, these data show that IL-36 has potent antitumor effects across preclinical models, representing a spectrum of human tumors that respond differently to immunotherapies, hinting at a fundamental role of this pathway in the TME.

To rule out potential artifacts of constitutive IL-36γ expression, we generated B16F10 cells with an inducible IL-36γ expression. Consistent with previous results, doxycycline-induced expression of IL-36γ led to significant control of already-established tumors (Figure 1B). To further validate antitumor effects of IL-36γ in a therapeutic context, we intratumorally administered either adeno-associated virus–expressing (AAV-expressing) IL-36γ or recombinant mouse (rm) IL-36γ protein and observed significant reduction in tumor growth (Supplemental Figure 1D and Figure 1C). Importantly, when grown in IL-36R–deficient (IL-36R–KO) mice, no difference in tumor growth was observed between IL-36γ–expressing and control cells (Figure 1D and Supplemental Figure 1E). Taken together, these results demonstrate that enhanced IL-36 signaling, even in the context of immune barren and immune-suppressed TMEs, can exhibit robust antitumor effects that are dependent on IL-36/IL-36R signaling in host cells.

IL-36R is reported to be expressed on both hematopoietic and nonhematopoietic cells, including innate and adaptive immune cells, stromal cells, and tumor cells (10). Therefore, we performed bone marrow chimera experiments to tease apart cellular components driving IL-36γ–mediated antitumor responses in our studies. Mice reconstituted with WT hematopoietic cells inhibited the growth of IL-36–overexpressing tumor cells (Figure 1E), but mice reconstituted with IL-36R–KO hematopoietic cells failed to do so. Interestingly, in 50:50 WT/IL-36R–KO mixed chimera, even 50% of WT hematopoietic cells was sufficient for strong antitumor immunity. These results demonstrate that hematopoietic cells responding to IL-36γ signaling are the drivers of antitumor responses. Moreover, mice that had rejected primary IL-36γ–expressing MC38 tumors, when rechallenged with parental MC38 cells after 2 months of being tumor free, exhibited enhanced effector T cell responses and rejected the tumors completely, suggesting establishment of protective immunological memory (Figure 1F and Supplemental Figure 1F). Collectively, these data demonstrate that IL-36 signaling in hematopoietic cells controls tumor growth by inducing potent antitumor immunity and protective memory responses.

### Enhanced IL-36γ activity within the TME affects both innate and adaptive immune cells.

**Figure 2:**
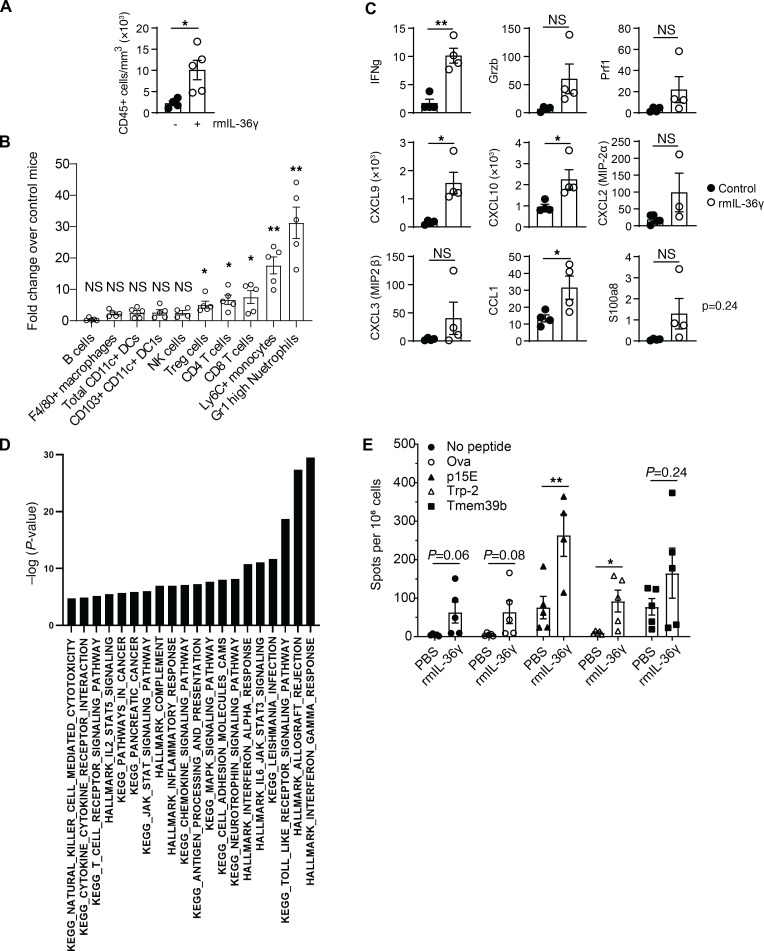
Enhanced IL-36 within the TME affects innate and adaptive immune cells. Analyses of B16F10 tumors after 10-day intratumoral administration of vehicle control (*n* = 5) or 25 μg/kg of rmIL-36γ (*n* = 5) and harvesting 24 hours after the last dose. (**A**) FACS analysis showing increased CD45^+^ immune cells. (**B**) Fold increase in the number of various immune cells in rmIL-36γ–treated B16F10 tumors. Fold increase was calculated by dividing the average numbers of individual cell types in rmIL-36–treated mice with the numbers of corresponding control mice. (**C**) Nanostring analysis showing select proinflammatory and effector molecule transcripts. The *y* axis represents raw counts of the indicated transcript. (**D**) Ingenuity Pathway Analysis of differentially expressed genes in Nanostring data set showing biological pathways that are significantly enriched in rmIL-36γ–treated B16F10 tumors compared with control tumors. *P* values were calculated using gene set enrichment analysis (GSEA). (**E**) ELISPOT analysis of splenocytes from B16F10 tumor-bearing mice treated with vehicle control (*n* = 5) or rmIL-36γ (*n* = 5). IFN-γ production upon culture with neoantigen peptides p15E, Trp2, and Tmem39b or irrelevant peptide OVA is shown. Data are shown as mean ± SEM. Unpaired 2-tailed Mann-Whitney *t* test. *P < 0.05, **P < 0.01. Data are representative of 2 independent experiments.

To understand the effect of IL-36 signaling on various hematopoietic cells in the TME, we administered rmIL-36γ intratumorally into B16F10 tumors and performed pharmacodynamic analyses. We first focused on the cellular assessment by flow cytometry (Supplemental Figure 2A) and observed a significant increase in overall CD45^+^ immune cell infiltration (Figure 2A). All major T cell subsets were increased and exhibited a pronounced activated phenotype (Figure 2B and Supplemental Figure 2B). Both cDC1 and cDC2 subsets, as well as F4/80^+^ macrophages, were increased, and most of them showed an increase in their activation state upon IL-36R activation (Supplemental Figure 2, C and D). We also observed a nonsignificant increase in NK cell numbers but not B cells numbers (Figure 2B). However, notably, the most robust impact was a 20- to 30-fold higher infiltration of Ly6C^+^ monocytes and Gr1^hi^ neutrophils into the TME of IL-36γ–treated tumors compared with untreated tumors (Figure 2B and Supplemental Figure 2, E and F). Next, to understand the consequences of these cellular effects, we performed Nanostring gene expression analysis using total RNA from rmIL-36γ–treated and untreated tumor homogenates. We found a significant increase in proinflammatory cytokines and chemokines (*Ifng, Granzyme B, CXCL9, CXCL10, CCL1, s100a8*) and signs of enhanced antigen presentation (*Tap1, Tapbp*) and type I IFN gene signature (*Ifit1, Ifit3*) (Figure 2C and Supplemental Figure 2, G and H). Elevated levels of selected cytokine and chemokines were also confirmed at the protein level by cytometric bead array assay (Supplemental Figure 2I). As expected, pathway analysis of differentially expressed genes in the TME indicated an enhanced MAP kinase signaling pathway and increased Th1 responses upon rmIL-36γ treatment (Figure 2D) (11). Enhancement of TLR stimulation/myeloid activation, antigen processing and presentation, JAK/STAT pathways, cytokine responses, and other proinflammatory pathways were observed, suggesting involvement of innate immune responses (Figure 2D). To identify de novo priming effects, we performed an enzyme-linked immunosorbent spot (ELISPOT) analysis and observed increased neoepitope peptide-specific T cells, suggesting antigen-specific T cell responses (Figure 2E). However, we observed increased IFN-γ–expressing cells, even in the absence of any peptide or in the presence of an irrelevant peptide (OVA) in tumors with rmIL-36γ, suggesting a nonspecific increase in the abundance of IFN-γ–expressing cells, such as activated T and NK cells in the TME with enhanced IL-36 signaling. These results suggest that IL-36 leads to broad activation of immune system in the TME.

Next, we used bone marrow chimera system to assess whether the increased intratumoral immune cell infiltration was cell intrinsic or dependent on nonhematopoietic cells. We first transferred WT, IL-36R–KO, or a mix of WT and IL-36R–KO hematopoietic cells into irradiated WT mice and then implanted WT B16F10 tumors. Similar to our observations in nonirradiated hosts, innate immune cells, particularly Ly6G^+^ neutrophils, were the most prominently mobilized immune cells upon rmIL-36γ treatment in an IL-36R–dependent manner (Supplemental Figure 2J). While we did not observe a large increase in total CD4^+^ T cells or CD8^+^ T cells in this setting, Ki67^+^ proliferating T cells were increased with a dependency on IL-36R expression (Supplemental Figure 2J). Cumulatively, our results suggest a profound effect of IL-36 signaling on both innate and adaptive immune cells in the TME. Given the observed changes in innate cells, including a dramatic infiltration of neutrophils and monocytes upon rmIL-36γ administration, we focused on delineating the mechanistic impact of IL-36 signaling on innate immune cells in the TME. Effects of IL-36 in mediating the adaptive immune system have been described by previous studies (12, 19).

### IL-36 can control tumor growth in mice lacking adaptive immune compartment.

**Figure 3:**
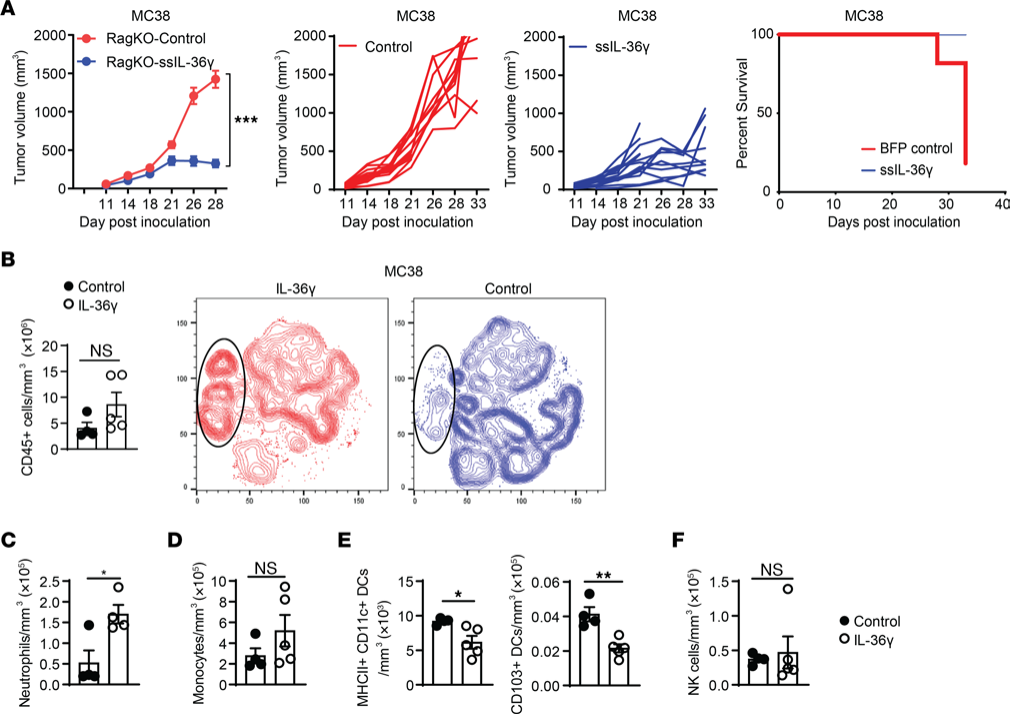
IL-36 regulates tumor growth in the absence of T and B cells. (**A**) Tumor growth of control or IL-36γ–expressing MC38 syngeneic tumors in Rag-KO mice. Tumor growth inhibition (left), individual tumor growth curves (middle), and survival curve (right) are shown (*n* = 10/group). (**B**) FACS analysis of showing increased CD45^+^ immune cells in the TME of control (*n* = 5) or IL-36γ–expressing MC38 tumors (*n* = 5) (left). tNSE plot analyses showing global changes in immune cell composition in the TME of MC38 tumors with IL-36γ expression (red) or without (blue). Substantially enhanced neutrophil population is shown in highlighted oval shape. (**C**–**F**) FACS analysis showing numbers for various innate immune cells in the TME of control (*n* = 5) or IL-36γ–expressing MC38 tumors (*n* = 5) in Rag-KO mice. (**C**) Neutrophils, (**D**) monocytes, (**E**) DCs, and (**F**) NK cells. Data are shown as mean ± SEM. (**A**) Two-way ANOVA followed by Šidák’s multiple comparison test. (**B**–**F**) Unpaired 2-tailed Mann-Whitney *t* test. *P < 0.05, **P < 0.01. Data are representative of 3 (**A**) and 2 (**B**–**F**) independent experiments.

To examine the contribution of innate immune cells in IL-36–mediated antitumor immune responses, we used Rag2-deficient (Rag-KO) mice that lack T cells and B cells. Remarkably, constitutive IL-36γ expression led to significant tumor control, even in the absence of T cells (Figure 3A). This result in Rag-KO mice is in line with what we observed in immunocompetent mice and, therefore, highlighted a major contribution of innate immune cells in the antitumor responses mediated by IL-36. Pharmacodynamically, we observed a trend of overall increase in CD45^+^ immune cells in IL-36γ–expressing tumors (Figure 3B). Again, neutrophils were the most abundantly increased immune cells and exhibited an activated phenotype, as exemplified by increased CD86 expression (Figure 3C and Supplemental Figure 3A). Monocytes showed a trend toward increased numbers (Figure 3D and Supplemental Figure 3B). DCs, including CD103^+^ DC1s were reduced in numbers, but with a trend of higher expression of CD86 (Figure 3E and Supplemental Figure 3, C and D). It is possible that these DCs migrated from tumor into the draining lymph node upon activation. We did not observe any increase in NK cells in this setting (Figure 3F). Taken together, these results strongly suggest that, even in the absence of adaptive immune cells, IL-36 can induce substantial tumor growth inhibition through its effects on innate immune cells.

### Neutrophils contribute significantly to IL-36–mediated antitumor responses.

**Figure 4:**
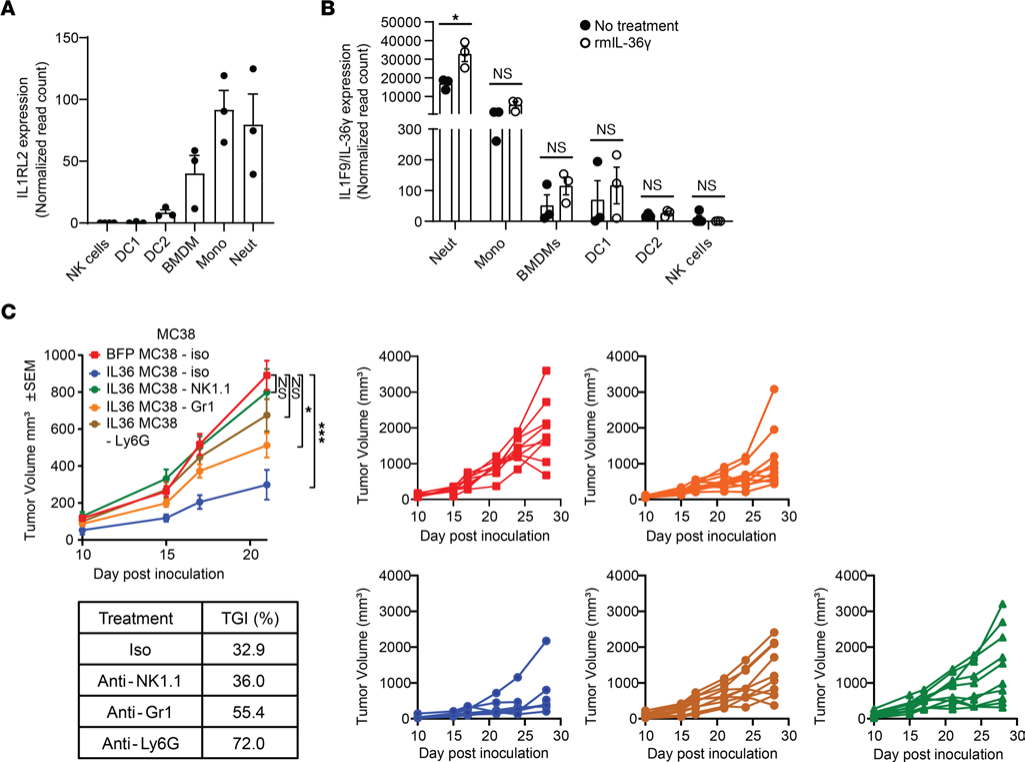
Neutrophils and NK cells contribute to IL-36–mediated antitumor responses in Rag-KO mice. (**A**) IL-36R (*Il1rl2*) expression in various immune cells isolated from spleen (NK, DCs) or bone marrow (BMDMs, monocytes, neutrophils) of WT C57BL/6 mice, with the highest IL-36R content found in neutrophils. Normalized read counts from bulk RNA-Seq were used. (**B**) IL-36γ (*Il1f9*) expression in various immune cells isolated from spleen (NK, DCs) or bone marrow (BMDM, monocytes, neutrophils) of WT C57BL/6 mice either untreated or treated with 500 ng/mL rmIL-36γ for 6 hours. The *y* axis is split to show minimal expression in certain cell types. Normalized read counts from bulk RNA-Seq were used. (**C**) Tumor growth of control or IL-36γ–expressing MC38 syngeneic tumors in Rag-KO mice administered with various cell-depleting mAbs. Anti-NK1.1. mAbs were used to deplete NK cells, Ly6G mAbs were used to deplete neutrophils, and Gr-1 mAbs were used to deplete both neutrophils and monocytes. Tumor growth inhibition (TGI) and individual tumor growth curves are shown (*n* = 10/group). Data are shown as mean ± SEM. (**B**) Unpaired 2-tailed Mann-Whitney *t* test. (**C**) Two-way ANOVA followed by Tukey’s multiple comparison test. *P < 0.05, ***P < 0.001. neut, neutrophils; mono, monocytes. Data are representative of 1 (**A** and **B**) and 2 (**C**) independent experiments.

Next, we wished to identify the dominant innate immune mediator(s) responding to IL-36 signaling in the TME. Among the innate immune cells purified from spleen and bone marrow of WT mice (Supplemental Figure 4, A and B), IL-36R transcript was highest in monocytes and neutrophils (Figure 4A). Neutrophils also expressed the highest amounts of ligand (IL-36γ), which was further increased upon ex vivo treatment with IL-36γ, suggesting a positive feedback mechanism (Figure 4B). The high expression of IL-36R in neutrophils and monocytes indicated their possible direct involvement in IL-36–mediated antitumor responses. To delineate their contribution to tumor control in vivo, we utilized anti-Ly6G or anti-Gr1 mAbs to deplete neutrophils alone, or both neutrophils and monocytes, respectively in Rag-KO animals bearing IL-36γ–expressing or control MC38 tumors (Supplemental Figure 4, C and D). As expected, increased IL-36γ in the TME led to tumor growth inhibition, but this tumor control was significantly blunted upon neutrophil depletion (Figure 4C), suggesting a major contribution of neutrophils in IL-36γ–mediated antitumor responses in vivo. While we observed efficient and comparable depletion neutrophils with both anti-Gr1 and anti-Ly6G mAbs, anti-Gr1 mAb was unable to achieve complete monocyte depletion (Supplemental Figure 4, C and D). Furthermore, effects of anti-Gr1 in reversing IL-36–mediated tumor control were milder than anti-Ly6G mAb, suggesting a potential heterogeneity in monocyte functions in their response to IL-36 signaling and contribution to antitumor immunity.

Additionally, because NK cells have previously been implicated in IL-36–mediated antitumor immune responses (19, 20), we wanted to determine the impact of NK cell depletion with anti-NK1.1 mAb in our system. In line with results of previous reports, we observed NK cells to contribute to IL-36–mediated tumor control (Figure 4C). However, lack of IL-36R expression on naive NK cells (Figure 4A) and lack of their expansion or activation with higher IL-36 in the TME (Figure 3F) suggest that NK cells may be responding indirectly to IL-36–mediated proinflammatory effects in the TME, potentially driven by neutrophils. Collectively, these results demonstrate that neutrophils and NK cells are key innate immune populations mediating IL-36–driven tumor control.

### IL-36 signaling–activated neutrophils demonstrate potent antitumorigenic effector functions.

**Figure 5:**
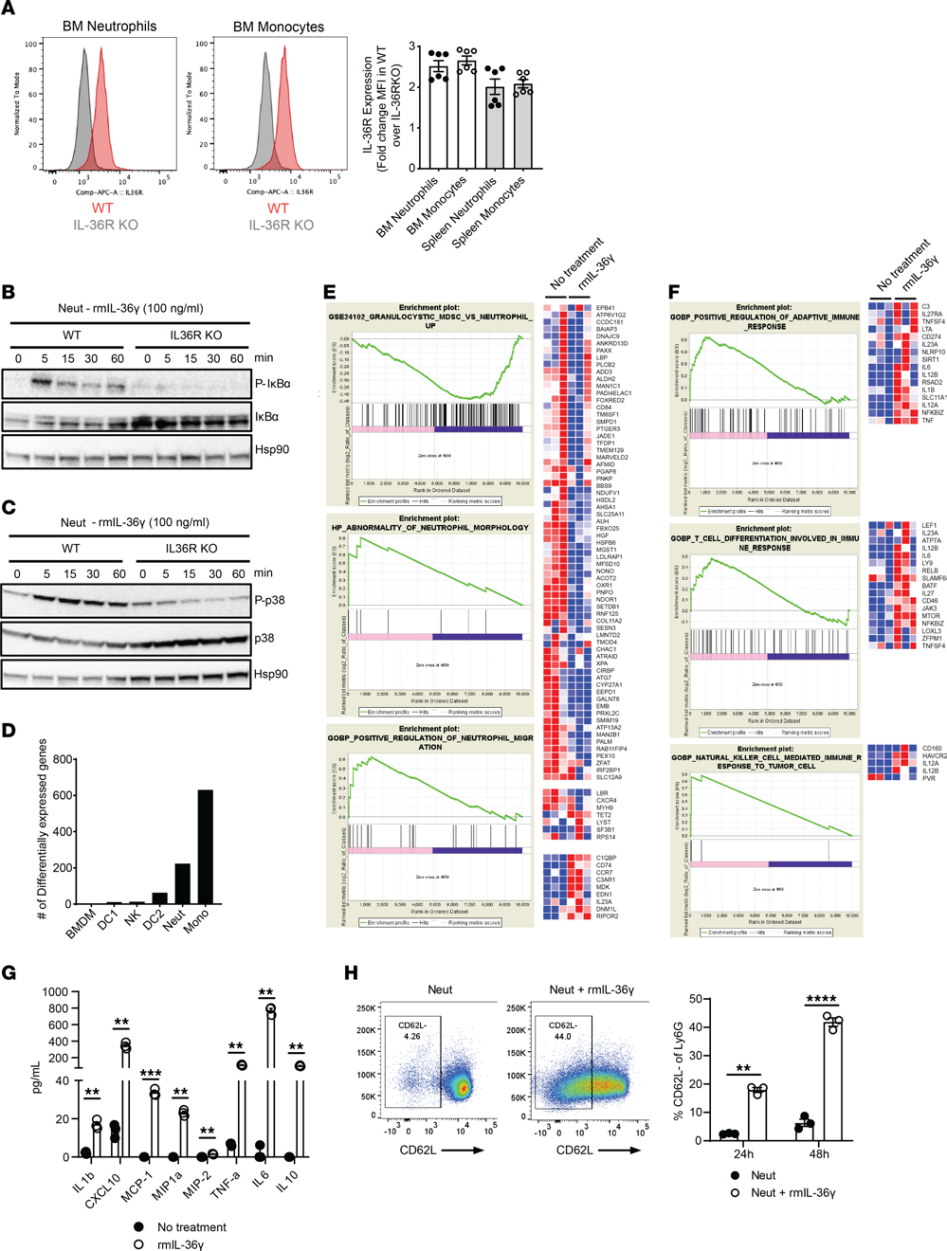
Cell autonomous IL-36 signaling is a potent activator of neutrophils. (**A**) IL-36R expression analysis on neutrophils and monocytes from bone marrow by flow cytometry using anti-IL-36R antibody (catalog 7501, ProSci). Cells were gated on CD11b^+^Ly6G^+^ for neutrophils and CD11b^+^Ly6C^+^ for monocytes. Histograms for WT and IL-36R–KO and mean florescence intensity (MFI) calculated from triplicates and 2 independent experiments is shown in the bar graph. MFI was normalized to that in KO cells. (**B** and **C**) Western blot analysis showing IL-36–mediated signaling in neutrophils treated with 100 ng/mL rmIL-36γ in WT and IL-36R–KO mice (**B**) NF-κB signaling. (**C**) p38 MAPK signaling. Hsp90 was used as loading control. (**D**) Number of differentially expressed genes in various innate immune cells isolated from spleen and bone marrow of WT C57BL/6 mice upon treatment with 500 ng/mL rmIL-36γ for 6 hours. (**E** and **F**) Pathway analysis of differentially expressed genes in neutrophils isolated from bone marrow of WT C57BL/6 mice upon treatment with rmIL-36γ for 6 hours showing pathways that (**E**) affect neutrophil-intrinsic biology and (**F**) modulate various other immune cells. (**G**) Production of proinflammatory cytokines and chemokines by neutrophils isolated from bone marrow of WT C57BL/6 mice and treated with 500 ng/mL rmIL-36γ for 24 hours. Cytokines and chemokines were detected using a multiplexed MSD kit. (**H**) IL-36–mediated activation of neutrophils, as depicted by change in CD62L expression with rmIL-36γ. Representative dot plots and average percentage positive cells are shown in the bar graphs. Data are shown as mean ± SEM. (**F** and **G**) Unpaired 2-tailed Mann-Whitney *t* test. ****P < 0.0001. Data are representative of 1 (**D**–**F**), 2 (**A**, **B**, **C**, and **G**), 3 (**H**) independent experiments.

Next, we investigated whether IL-36 acts directly on neutrophils to modulate their antitumor phenotype. We confirmed surface expression of IL-36R protein on neutrophils by flow cytometry (Figure 5A and Supplemental Figure 5A). IL-36 signaling is known to induce the canonical NF-κB and MAP kinase pathways (11), and consistent with it, rmIL-36γ–treated neutrophils showed robust induction of IκBα (Figure 5B) and p38 MAP kinase (Figure 5C) in WT but not IL-36R–KO neutrophils. Overall, these data demonstrate that a functional IL-36R is expressed on neutrophils, and IL-36γ ligand binding transduces cell-autonomous signaling.

To investigate functional consequences of IL-36R signaling in neutrophils, we performed bulk RNA-Seq analysis. Ex vivo treatment of purified neutrophils with rmIL-36γ resulted in nearly 200 differentially expressed genes (Figure 5D). This included a significant decrease in genes associated with suppressive functions of PMN-MDSCs, suggesting that IL-36 signaling can modulate tumor-infiltrating neutrophils to switch to an antitumor phenotype. We observed an increase in genes associated with neutrophil migration (Figure 5E), supporting our previous observation of high neutrophil influx to TME in IL-36 expressing tumors. Importantly, there was a concomitant increase in gene signatures associated with activation of adaptive T cell responses and activation of NK cells (Figure 5F). We also observed many differentially expressed genes in monocytes (Figure 5D), mainly those involved in cytokine-mediated communication between immune cells, granulocyte adhesion and diapedesis, and TREM-1 and Toll-like receptor signaling (data not shown). While this suggests an activated state of monocytes upon IL-36γ signaling, we did not pursue this further due to the lack of in vivo evidence for a critical role of monocytes in IL-36–mediated antitumor immunity (Figure 4C).

Consistent with RNA-Seq data, and akin to classical N1 neutrophils that exhibit antitumor phenotype (29), rmIL-36γ–treated neutrophils produced elevated levels of multiple proinflammatory cytokines and chemokines, including IL-6, IL-1b, and TNF in WT cells (Figure 5G), but not in IL-36R–KO neutrophils (Supplemental Figure 5B). High amounts of chemokine CXCL10 secreted by neutrophils upon IL-36R signaling (Figure 5G) support their role in mediating T cell infiltration in cold tumors. rmIL-36γ–treated neutrophils exhibited an activated phenotype, as evidenced by reduced CD62L (Figure 5H) and increased CD86 expression (Supplemental Figure 5C). Barring few differences, neutrophils isolated from B16F10 tumors also showed similar increase in many of the same proinflammatory cytokines and chemokines when treated ex vivo with rmIL-36γ (Supplemental Figure 5D), suggesting that bone marrow neutrophils can be used as a model for tumor-infiltrating neutrophils in investigating the effect of IL-36R signaling on their antitumor responses. Therefore, due to ease of isolation and greater abundance, we used bone marrow neutrophils for our subsequent experiments. Cumulatively, these data suggest that IL-36 signaling in neutrophils results in an activated, proinflammatory phenotype.

### IL-36–activated neutrophils can directly kill tumor cells.

**Figure 6:**
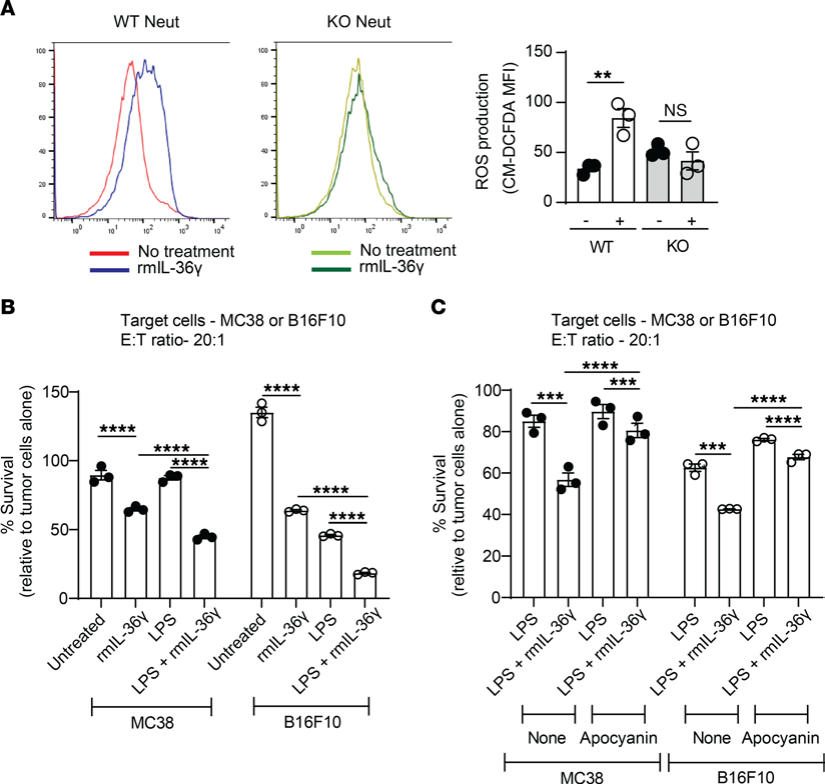
IL-36–activated neutrophils directly kill tumor cells. (**A**) Neutrophils isolated from WT or IL-36R–KO mice and either untreated (-) or treated with 500 ng/mL rmIL-36γ (+) showing ROS production, as detected by increased mean florescence intensity (MFI) of ROS-sensitive dye CM-DCFDA. Individual histograms (left) and average MFI (right) are shown. (**B**) Direct killing of luciferase-expressing MC38 cells or B16F10 tumor cells by WT neutrophils either untreated or treated with indicated reagents. rmIL-36γ (500 ng/mL) and/or LPS (100 ng/mL) were added to culture media for 48 hours. Data are represented as luciferase signal relative to target cells cultured without the presence of neutrophils. (**C**) Direct killing of luciferase-expressing MC38 cells or B16F10 tumor cells by WT neutrophils either untreated or treated with indicated reagents. rmIL-36γ (500 ng/mL) and/or LPS (100 ng/mL) were added to culture media for 48 hours. For ROS inhibition, neutrophils were treated 300 μM Apocynin for 1 hour before they were added to the culture. Data are represented as luciferase signal relative to target cells cultured without the presence of neutrophils. Data are shown as mean ± SEM. (**A**) Unpaired 2-tailed Mann-Whitney *t* test. (**B** and **C**) One-way ANOVA followed by Tukey’s multiple comparison test. **P < 0.01, ***P < 0.001, ***P < 0.0001. neut, neutrophils. Data are representative of 2 (**C**) and 3 (**A** and **B**) independent experiments.

Activated neutrophils have been described to kill tumor cells directly via induction of ROS and granzyme B (8). rmIL-36γ–treated WT neutrophils produced high levels of ROS compared with untreated cells, while IL-36R–KO neutrophils failed to do so (Figure 6A). This increase in ROS was further boosted by the presence of irradiated tumor cells, a condition mimicking dying cancer cells in the TME, which can potentially provide additional activation signals, such as TLR stimulation (Supplemental Figure 6A). Even though in a minor proportion of cells, we also observed significantly more granzyme B (Supplemental Figure 6B) upon rmIL-36γ treatment. Consequently, IL-36γ–treated neutrophils showed enhanced ability to directly kill variety of tumor cells, representing not just immunologically warm (MC38) but also cold tumors (B16F10, Pan02, KPC) (Figure 6B and Supplemental Figure 6C). The cytotoxic ability of IL-36γ–treated neutrophils was enhanced by cytokines, such as IL-1β, IL-2, or TLR ligands, such as LPS or irradiated tumor cells (Figure 6B and Supplemental Figure 6D). The increased cytotoxicity was observed, even when TGF-β levels were high, a condition representing highly immunosuppressive TME (Supplemental Figure 6D). Furthermore, when ROS production machinery was blocked by NADPH oxidase inhibitors like apocynin or diphenylene iodonium, we observed reduction in IL-36–mediated cytotoxicity (Figure 6C and Supplemental Figure 6E), suggesting that enhanced ROS production contributed to their cytotoxicity. However, survival of tumor cells was not completely restored, indicating additional mechanisms. Collectively, these results demonstrate that IL-36 signaling in neutrophils results in induction of potent antitumor effector functions capable of directly killing tumor cells.

### IL-36–activated neutrophils modulate responses of other immune cells in the TME.

**Figure 7:**
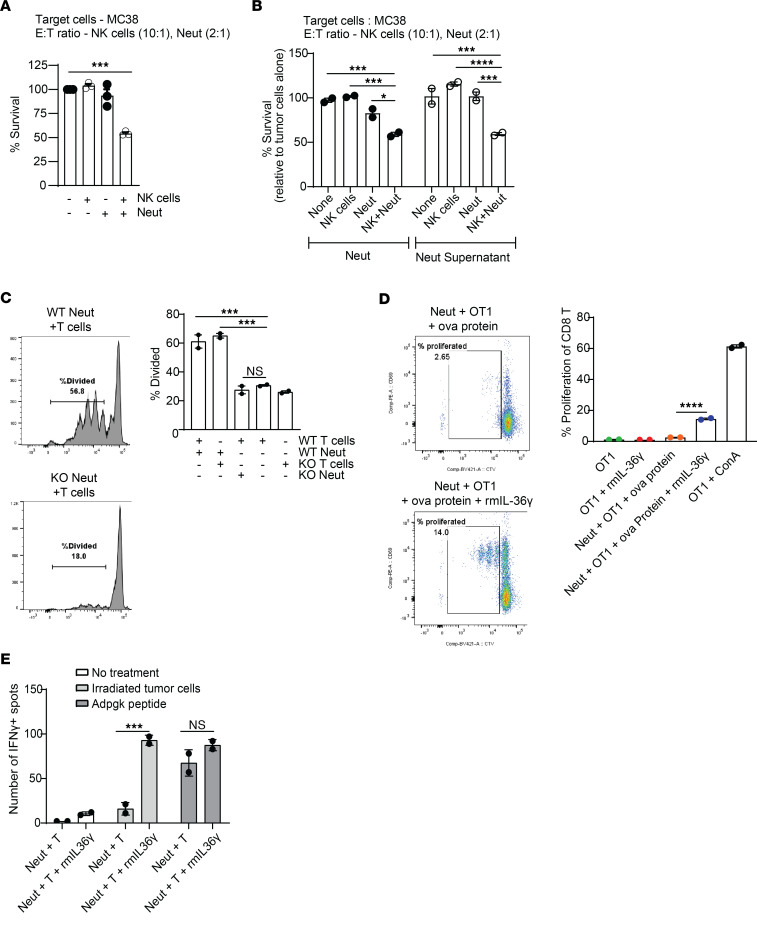
IL-36–activated neutrophils modulate NK cell cytotoxicity and T cell proliferation. (**A** and **B**) Cell killing of MC38 cells expressing luciferase (MC38-Luc) by (**A**) NK cells alone, neutrophils alone or combination of NK cells, and neutrophils when treated with 500 ng/mL rmIL-36γ or (**B**) NK cells alone, supernatant from IL-36–treated neutrophils alone, or combination of NK cells and supernatant. The E:T ratio for NK cells was 10:1 and for neutrophils it was 2:1. Data are represented as luciferase signal relative to MC38-Luc cells cultured alone, without the presence of NK cells and neutrophils. (**C**) Anti-CD3/CD28 stimulated proliferation of WT or IL-36R–KO T cells cocultured with either WT neutrophils or IL-36R–KO neutrophils for 72 hours. All conditions contained 500 ng/mL rmIL-36γ and 1 μM of CpG class C. Representative histograms are shown on the left, and average proliferation is shown in the bar graph on right. (**D**) OT-1 T cell proliferation for 48 hours in the presence of bone marrow neutrophils that were previously either untreated or prestimulated with 500 ng/mL of rmIL-36γ for 2 h and then fed with OVA protein. Representative graphs are shown on the left and average proliferation is shown in the bar graph. ConA is used as positive control for T cell proliferation. (**E**) IFN-γ ELISPOT assay using splenocytes from MC38 tumor-bearing mice cocultured with neutrophils that were previously either untreated or treated with 500 ng/mL rmIL-36γ for 2 hours and fed with MC38 tumor cell lysate or neoantigen Adpgk peptide. Number of IFN-γ–positive spots after 24 hours is shown. Data are shown as mean ± SEM. (**E**) Unpaired 2-tailed Mann-Whitney *t* test. (**A**–**D**) One-way ANOVA followed by Tukey’s multiple comparison. *P < 0.05, ***P < 0.001, ****P < 0.0001. neut, neutrophil. Data are representative of 2 (**C**–**E**) and 3 (**A** and **B**) independent experiments.

To ascertain whether IL-36–activated neutrophils modulate responses of other immune effector cells in the TME, we started by examining NK cells, which we had found to contribute to IL-36γ–driven tumor inhibition in Rag-KO animals but lack IL-36R in their naive state (Figure 4, A and C). We hypothesized that neutrophils modulate NK cell cytotoxicity. To address this, we assessed tumor cell killing by NK cells in the presence or absence of IL-36–treated neutrophils. Unlike previous assay conditions (Figure 6B), where we had used an E:T (neutrophils/tumor cells) ratio of 20:1, we reduced the E:T ratio to 2:1 to avoid maximal neutrophil-mediated killing. As hypothesized, naive NK cells alone were unable to kill tumor cells upon rmIL-36γ treatment (Figure 7A). However, their cytotoxicity was significantly enhanced when cocultured with neutrophils and rmIL-36γ (Figure 7A and Supplemental Figure 7A), suggesting that NK cell–mediated tumor control observed in Rag-KO mice might be indirectly driven by proinflammatory functions of IL-36–activated neutrophils. Because NK cell cytotoxicity is known to be enhanced by secreted factors, such as IL-2, IL-15, and TNF, we speculated that the modulation by IL-36–treated neutrophils could be mediated by cytokines and chemokines produced by them. Indeed, addition of supernatant from rmIL-36γ–activated neutrophils to NK cells resulted in enhanced NK cytotoxicity (Figure 7B).

Our RNA-Seq analysis indicated that IL-36 treatment leads to downregulation of immune-suppressive functions in neutrophils (Figure 5E). TLR stimulation, particularly TLR7/8/9 stimulation, is shown to overcome suppression of T cell proliferation by tumor-infiltrating neutrophils (30, 31). In line with those observations, TLR stimulation reversed the suppressive functions of neutrophils and led to significant induction of T cell proliferation, and importantly, IL-36γ treatment further augmented T cell proliferation in the presence of WT neutrophils but not with IL-36R–KO neutrophils (Figure 7C and Supplemental Figure 7, B and C). This was not due to IL-36 signaling in T cells themselves, since WT neutrophils were able to induce equivalent proliferation in both WT and IL-36R–KO T cells (Figure 7C). It is noteworthy that IL-36γ treatment alone was not sufficient to overcome neutrophil-mediated suppression of T cell proliferation in vitro and required TLR stimulation (Supplemental Figure 7B). Furthermore, while we observed significant enhancement of T cell proliferation in presence of WT neutrophils compared with IL-36R–KO neutrophils, there was no difference in T cell proliferation induced by WT neutrophils when external rmIL-36γ was added compared with conditions where no external rmIL-36γ was added, especially when TLR agonists were present (Supplemental Figure 7C). We hypothesized that neutrophils might be producing IL-36γ upon TLR stimulation, which in turn could activate neutrophils. Indeed, we found higher levels of IL-36γ in cultures treated with TLR agonists (Supplemental Figure 7D). Even IL-36R–KO neutrophils produced almost equal amounts of IL-36γ with TLR agonists compared with WT neutrophils, while only WT neutrophils augmented T cell proliferation (Figure 7C and Supplemental Figure 7B). This suggests that IL-36R signaling in WT neutrophils provides additional signals to enhance T cell proliferation, either through cell-cell contact or secreted factors, which needs further investigation. In this regard, neutrophil-mediated enhancement of T cell proliferation via secreted factors has been described previously (32). Overall, our results support a functional consequence of IL-36 signaling in neutrophils in controlling tumor growth through induction of T cell proliferation under appropriate conditions.

Emerging literature suggests that certain stimuli induce neutrophils to function as antigen-presenting cells (33, 34). We found that IL-36γ–activated neutrophils exhibited a shared signature with WT DCs as well as enrichment of a signature associated with positive regulation of adaptive immune responses (Supplemental Figure 8A and Figure 5F), implying a potential role in antigen presentation. In line with this, rmIL-36γ–treated neutrophils pulsed with OVA protein induced OT-1 proliferation, while untreated neutrophils failed to do so (Figure 7D). Even though, IL-36–treated neutrophil-induced T cell proliferation was lower compared with T cell proliferation induced by concanavalin A (ConA), it was significantly higher than that with untreated neutrophils. Additionally, we observed increased OVA uptake by rmIL-36γ–treated neutrophils (Supplemental Figure 8B). These data suggest that high antigen burden upon IL-36γ treatment could overcome some of the suppression by neutrophils in antigen presentation, providing a potential mechanistic basis for higher OT-1 T cell proliferation. To rule out the possibility that a minor proportion of contaminating cells in our enriched neutrophil population contributed to this phenotype, we sorted neutrophils to 100% purity and observed a similar increase in induction of OT-1 T cell proliferation by rmIL-36γ–treated neutrophils, demonstrating cell autonomous effects in neutrophils (Supplemental Figure 8C). To further test our hypothesis, IL-36γ–treated neutrophils were incubated with irradiated MC38 tumor cells and T cell stimulation was assessed by ELISPOT. We, again, observed that IL-36γ–treated neutrophils enhanced IFN-γ production by tumor antigen-specific T cells isolated from MC38 tumor-bearing mice (Figure 7E). Conversely, when neutrophils were pulsed with a MC38 neo-antigen-specific peptide, Adpgk, which does not need processing for presentation on the surface, there was no difference in T cell proliferation induced by IL-36γ–treated neutrophils compared with that of untreated neutrophils. This implies that IL-36γ–treated neutrophils can process and present antigens. Even though we did not observe enrichment of the antigen presentation pathway in our bulk RNA-Seq data, the enrichment of DC-like signature in IL-36γ–treated neutrophils, taken together with the experimental results presented here, suggests that IL-36γ could induce antigen processing and presentation by neutrophils, which in turn can lead to enhanced adaptive immune responses. Additionally, IL-36γ treatment led to an increase in the frequency CD103^+^ and CD40^+^ neutrophils (Supplemental Figure 8D) as well as an increase in the expression of SIINFEKL-loaded MHC class I on Act-mOva transgenic neutrophils (35) (Supplemental Figure 8E). Even though our data showed the potential of IL-36γ–treated neutrophils to process and present antigens, we cannot rule out the possibility of contribution of few contaminating cells to this phenotype, and further investigation is needed to tease apart the potential mechanism. Collectively, our results show that IL-36 acts directly on neutrophils and, in concert with additional signals within the TME, can modify their phenotype to antitumor effector cells resulting in tumor control.

**Figure 8:**
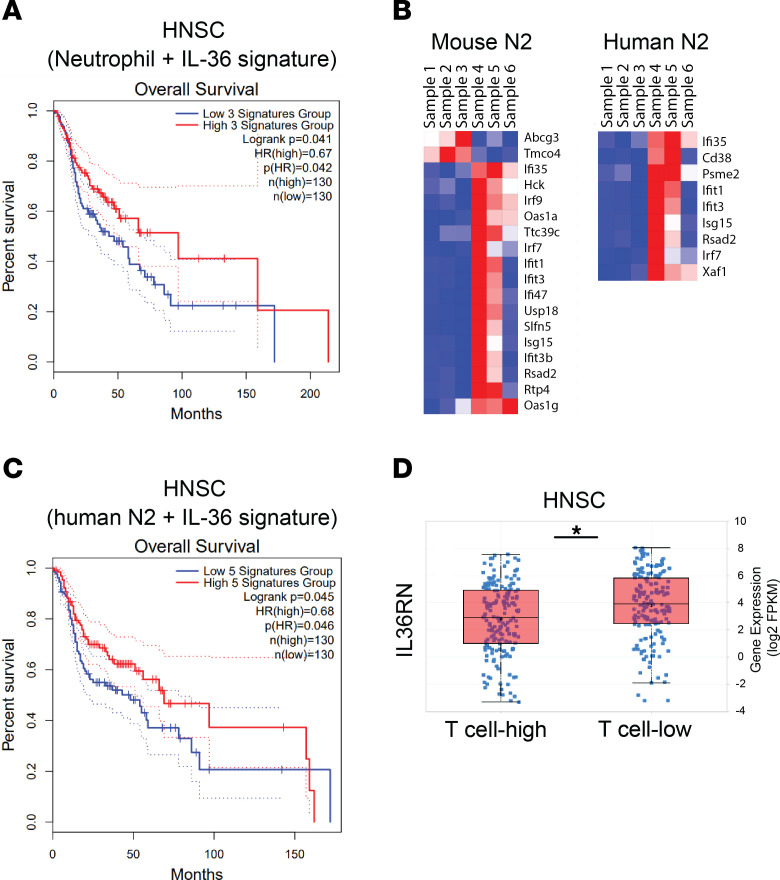
Enhanced IL-36 signature combined with neutrophil signature predicts better survival in patients with cancer. (**A**) Kaplan-Meier curve showing overall survival of patient subsets with high versus low neutrophil and IL-36 signature in head and neck carcinoma (HNSC). (**B**) Shared mouse and human N2 neutrophil subset genes that are significantly enriched in rmIL-36γ–treated neutrophils. Samples 1–3 represent 3 replicates of untreated mouse neutrophils and 4–6 represent 3 replicates of neutrophils treated with 500 ng/mL rmIL-36γ for 6 hours. (**C**) Kaplan-Meier curve showing overall survival of patient subsets with high versus low neutrophil N2 and IL-36 signature in head and neck carcinoma. (**D**) TCGA analysis showing higher expression of IL-36RN associated with lower number of T cells in head and neck carcinoma.

Next, we assessed the potential effect of the IL-36/IL-36R axis on neutrophils in human tumors and determined the added prognostic benefit, if any, of high IL-36 signaling in a neutrophil-rich immunosuppressive TME that is representative of hard-to-treat tumors. Survival analysis of TCGA data showed that higher IL-36 signature was able to improve prognosis, even in the context of neutrophil-enriched immunosuppressive environment (Figure 8A and Supplemental Figure 9, A and B) and that higher neutrophil signature in the context of higher IL-36 signature provides the highest survival benefit (Supplemental Figure 9B). In this regard, it has been reported that, while MDSCs and neutrophil signatures in the TME are negative prognostic factors, high IL-36α expression is associated with better prognosis (36). Recent single-cell studies on mouse and human neutrophils have uncovered significant heterogeneity in neutrophil populations that are enriched in a tissue- and disease-specific context (37, 38). While IL-36γ treatment turned on diverse transcriptional programs representing a continuum of states of several subsets (Supplemental Figure 9C), we found a greater overlap with the gene signature of a small but distinct neutrophil subset reported in humans and mice with type I IFN response gene signature (Figure 8B and Supplemental Figure 9C, hN2 and mN2, as described in ref. 37). This subset has been reported to be associated with worse survival in lung adenocarcinoma. However, when combined with higher IL-36 signature, it significantly improved survival (Figure 8C and Supplemental Figure 9D), again suggesting that IL-36 assists in overcoming and reversing an immunosuppressive TME. IL-36R is known to constitutively bind an antagonist ligand, IL-36RN, that needs to be replaced by 1 of the 3 activating ligands for downstream signaling (11). IL-36RN is highly expressed across human tumors in the TCGA data set (data not shown), suggesting that tumors actively suppress IL-36R signaling within the TME. Accordingly, higher expression of IL-36RN is associated with low T cell infiltration (Figure 8D and Supplemental Figure 9E). Collectively, these data suggest a potential role of IL-36R signaling in modulating human tumor outcomes. Based on our cumulative data, we propose a model where IL-36 signaling in multiple cell types contributes to enhanced antitumor immunity, but its modulation of neutrophil phenotypes is likely to play a central and multifaceted role in mounting a comprehensive antitumorigenic immune response. IL-36–treated neutrophils not only kill tumor cells directly, but also modulate functions of several key cell types, including T cells, NK cells, and potentially DCs to limit tumor growth.

## Discussion

Even though a considerable focus of cancer immunotherapy has been T cells and adaptive immune cells, the ability to engage both innate and adaptive cells is likely a key determinant particularly in eradication of established tumors (6). Here, we showed that IL-36 is a potent activator of innate immune cells and complement adaptive immunity to mediate strong antitumor responses. Furthermore, we found that IL-36 signaling in neutrophils modifies them into proinflammatory cells that modulate T and NK cell responses. We systematically characterized the functional consequences of IL-36 signaling in neutrophils and demonstrated that IL-36–treated neutrophils can directly kill tumor cells, induce cytolytic activity in NK cells, and enhance T cell proliferation. This crosstalk between IL-36–treated neutrophils and rest of the immune compartment in the TME results in mounting a highly potent antitumorigenic response.

Our findings extend those of previous reports describing the effect of IL-36 on tumor control (19–22) and demonstrate that responses are primarily driven by hematopoietic cells. While our observations of enhanced adaptive immunity upon increased IL-36 expression in the TME are also consistent, we show robust expansion of innate immune cells, particularly neutrophils. A previous study found that neutrophils increased with increasing IL-36 mRNA expression in MC38 tumors (20); however, in contrast to the findings in our study, the role of neutrophils on antitumor responses was not pursued due to incomplete depletion of neutrophils. Our observation of functional IL-36R on neutrophils validates recent results reported in inflammatory conditions (14, 15). NK cells have been found to contribute to IL-36–mediated tumor control in vivo (19). Interestingly, even in that study, IL-36γ–induced IFN-γ production by NK cells has been observed only in the presence of IL-2 (19). Here, we show that IL-36γ alone is unable to activate naive NK cells. However, neutrophils responding to IL-36γ could modulate NK cell cytotoxicity, suggesting that NK cell–mediated responses in vivo could be secondary effects derived from IL-36 activation in other immune cells, including neutrophils. This is supported by the observation that Rag-KO mice with intact NK cells, but depleted of neutrophils, failed to control tumor growth. However, it is also possible that proinflammatory signals such as IL-2 in WT mice induce a functional IL-36R expression on NK cells, thus enabling them to respond to IL-36 ligands in the TME. It has also been reported that γδT cells are increased in the TME upon IL-36 expression and can respond to IL-36 (19). While we did not test or observe this directly in our studies, it is interesting that neutrophils have been shown to influence tumoricidal activities of γδT cells via induction of cytotoxic mediators (39), a scenario likely to occur with enhanced IL-36 expression in the TME. A previous study (19) had observed a minimal impact of intratumoral IL-36 expression in Rag-KO mice, but Rag2/IL2Rg double-deficient mice, which lack not only T and B cells but also innate lymphoid cells (ILCs), were used in that study. In this regard, it was recently shown that IL-36R signaling regulates IL-23–driven IL-22 production potentially by ILCs, which was associated with significantly lower neutrophils in the intestines of *Citrobacter rodentium–*infected IL-36R–KO mice (40). Therefore, it is possible that, in Rag-KO mice with IL-36γ–expressing tumors, ILCs produce factors to recruit neutrophils, whereas Rag2/IL2Rg double-deficient mice fail to do so. Additionally, because we observed milder tumor control with tumor cells expressing IL-36γ without an ectopic signal sequence (Supplemental Figure 1C), it is also possible that the expression of IL-36 is lower in their model. Further studies are needed to tease apart these possibilities.

Neutrophils constitute one of the most important cellular components of innate immunity, playing an indispensable role in host defense (41, 42). However, the role of neutrophils in cancer is contentious, with both tumor-promoting and tumor-controlling functions described, depending on the tumor type, the TME, and the presence of a constellation of effectors and immune-modulating factors (39, 43). While tumor-associated neutrophils or PMN-MDSCs are considered immunosuppressor cells and are generally associated with nonfavorable outcomes, exceptions have been observed, and dual roles of neutrophils in tumor biology are increasingly appreciated (43). Neutrophils have been shown to perform diverse biological functions, some of which can potentially mediate antitumor response (41, 42). For instance, they leave trails for antigen-specific CD8 T cells to infiltrate airways via the production of chemokines in influenza (44). Our data show that agents such as IL-36 have the potential to induce T cell–attracting chemokines by neutrophils and potentially convert immunologically cold tumors to hot tumors. Moreover, in concert with appropriate signals such as TLR stimulation, IL-36 signaling in neutrophils enhances T cell proliferation and modulates NK cell functions. Future studies to identify such factors might be important to leverage the full therapeutic potential of IL-36 and its effects on neutrophils and PMN-MDSCs in mounting antitumor immune responses.

While we demonstrated and highlighted the importance of neutrophils and other innate immune cells in IL-36–mediated antitumor immune responses, we did not address the contributions of T cells to the efficacy, primarily since this has already been documented (19–22). Our observation that IL-36 expression in MC38 tumors in WT mice results in complete tumor rejection but only delays tumor growth in Rag-KO mice is consistent with previous reports and supports the contribution of T cells (19). This also confirms the findings that engaging both adaptive and innate immune cells is required for complete eradication of large, established tumors (6).

Even in human tumors, neutrophils or PMN-MDSCs are one of the most abundant cell types (42). Recent studies have demonstrated considerable plasticity and polarization of neutrophils under various conditions (45). Therefore, therapeutic exploration and exploitation of neutrophils to combat tumors is an underrepresented, yet promising and attractive, field of research (42). While certain agents have been shown to modify neutrophil functions in the TME, inadequate understanding of the mechanisms by which neutrophils act to promote or inhibit tumor growth has limited the development of novel therapeutics. We describe what we believe to be novel biology of cell-autonomous impact of IL-36–driven antitumorigenic effects of neutrophils/MDSCs. The prognostic benefit of high levels of endogenous IL-36 in tumors with higher neutrophil signature suggests that this effect can be further enhanced with exogenous IL-36 treatment. Further studies to explore and leverage the biology of the IL-36/IL-36R axis in human neutrophils can guide novel anticancer therapies.

## Methods

### Animals.

We used 5- to 9-week-old WT C57BL/6 animals from Charles River Laboratories and 6- to 10-week-old RAGN12-KO (Rag-KO) mice from Taconic. IL-36R–KO mice (28) and CAG-OVAL mice (35) are described previously.

### Cell lines.

All parental syngeneic tumor cell lines were purchased from ATCC and cultured according to recommended growth media containing 10% FBS (Hyclone) and L-glutamine at 37°C in 5% CO_2_ in a humidified incubator. KPC and IL-36 reporter cell lines were generated in-house as described before (11, 46). Authentication of tumor cell lines was performed by short tandem repeat profiling through IDEXX BioAnalytics, with additional mycoplasma contamination testing for in vivo studies.

Tumor cell lines with IL-36γ overexpression were generated using retroviral transduction. Briefly, GP2-293 packaging cell lines (Takara) were transfected with IL-36γ–pMSCV BFP vector with or without CD8a signal sequence, using Lipofectamine (Thermo Fisher Scientific) to generate retroviruses according to the manufacturer’s guidelines. Tumor cells were transduced by spinoculation, sorted based on BFP after 48 hours, expanded, and used for downstream experiments.

For the inducible cell lines, plasmid was generated with Tet-on promotor-IRES-GFP in between ROSA26 homology arms at the 3′ and 5′ end of the construct, and IL-36 was cloned before IRES. Cells underwent a CRISPR knockin transfection with linearized plasmid and gRNA targeting of ROSA26 and were incubated for 3 days before 250 ng/mL doxycycline was added to the cells overnight. Cells were then sorted for GFP and target expression to generate a stable inducible cell line. All cell lines were maintained at low passage numbers in complete DMEM media, supplemented with 10% HyClone FBS (Cytvia Hyclone), 1% Penicillin-Streptomycin (Gibco, 15140122), and 1% Glutamax (Gibco, 35050061).

### Murine IL-36γ generation.

Murine IL-36γ (amino acids 13–164, Amgen) was cloned as an N-terminal His6, SUMO fusion in pET28, and transformed into *E*. *coli* BL21(DE3). Expression was induced with IPTG at 37°C in LB media supplemented with glucose. Cells were harvested by centrifugation and lysed with a microfluidizer in buffer (50 mM Tris, pH 7.5; 500 mM NaCl; 5 mM imidazole). Purification was carried out with samples on ice or 4°C. Lysate was clarified by centrifugation, and rmIL-36γ was captured by immobilized metal affinity chromatography (Ni-NTA, Qiagen). The column was washed with 10 cv of 4 M urea in lysis buffer to reduce endotoxin followed by 20 cv of lysis buffer supplemented with 10 mM imidazole. Protein was eluted with a linear gradient of imidazole and fractions containing rmIL-36 were pooled. SUMO tag was removed by treatment with SUMO protease. Tag-free protein was further purified by reverse IMAC and size exclusion chromatography into formulation buffer (PBS).

### In vivo tumor studies.

Cultured tumor cells were dissociated into single cells with 0.05% Trypsin-EDTA (Gibco), and 3 × 10^5^ tumor cells (MC38, Renca, CT26) or 2 × 10^5^ B16F10 tumor cells were injected subcutaneously in 100 μL serum-free media into the right hind flank of mice. Animals were randomized into control or treatment groups if they were being treated when tumors were approximately 100 mm^3^. Tumor volume measurement was performed twice per week and calculated as *V* = (length × with × height) mm^3^. Studies were terminated when tumor volume reached 2,000 mm^3^ or twice the median survival of control mice, unless otherwise shown. Tumor growth inhibition was calculated on the day the first animal reached a maximum tumor volume of either 2,000 mm^3^ in the absence of treatment or 800 mm^3^ with treatment.

For bone marrow chimera, recipient CD45.2^+^ mice were X-ray irradiated (MultiRad X-Ray Irradiator) using 2 doses for total of 7 Gy. Donor bone marrow from WT CD45.1^+^ congenic mice or IL-36R–KO CD45.2^+^ mice was harvested and mixed at a 100%:0%, 0%:100%, and 50%:50% ratio. After irradiation, recipient mice were engrafted with 3 × 10^6^/200 μL donor bone marrow mixture by retro-orbital injection. After 8~10 weeks, fully reconstituted mice were implanted with indicated cell type (B16F10 or MC38), and tumors were harvested at the indicated time point or continued to monitor tumor growth.

For AAV experiments, mice were randomized, and either AAV-expressing GFP or IL-36γ was administered intratumorally (5 × 10^12^/mouse) in a total volume of 50 μL, 3 times per week.

### Tissue processing and flow cytometry.

Single-cell suspensions were prepared from harvested tumor, spleens, and lymph nodes and stained as described previously (46). For IL-36R analysis, bone marrow cells and splenocytes were stained with primary anti-IL-36R antibodies (5A5, Invitrogen, 38013, 1:100 or Prosci, 7501, 1:100) followed by secondary anti-rabbit antibody (1:200). Antibodies used for all flow cytometry analysis are described in Supplemental Table 3.

### Depletion studies in Rag-KO animals.

Depleting antibodies or their corresponding isotype controls were administered intraperitoneally starting on the day of tumor implantation and continued twice per week dosing up to 24 hours prior to last tumor measurement, for a total of 7 doses. For NK cells, anti-NK1.1 (BioXCell, BE0036) mAb was used for an initial dose of 500 μg/mouse and maintenance dose of 250 μg/mouse. For neutrophils, anti-Ly6G (BioXCell, BE0075-1) was administered at 250 μg/mouse, and for neutrophil and monocyte depletion, anti-Gr1 mAb (BioXCell, BE0075) was dosed at 250 μg/mouse. Corresponding isotype controls from BioXCell were used (BE0085, BE0089, BE0090).

### Nanostring analysis.

Total RNA extracted from snap-frozen tumor samples using a RNeasy kit (Qiagen) was analyzed for gene expression analysis with a nCounter Pan Cancer IO 360 panel (Nanostring Technologies). Pathway analysis for differentially expressed genes was done with the Hallmark and Curated KEGG gene sets on Molecular Signatures Database (MSigDB) with FDR *q* cutoff of less than 0.05 (47, 48).

### Cell-type specific isolations.

For cell-specific isolations and in vitro assays, spleens were flushed with RPMI media and dissociation buffer, followed by tissue homogenization, filtering, and RBC lysis using ACK buffer. Bone marrow were processed as described previously (48). Neutrophils were isolated from bone marrow using the EasySep mouse neutrophil enrichment kit (STEMCELL Technologies, 19762). For sorting, enriched neutrophils were stained with Ly6G-PE (Biolegend, 127607) and Sytox Blue (Thermo Fisher Scientific, S34857). Live and Ly6G^+^ cells were sorted on a BD FACSAria Fusion Flow Cytometer (BD Biosciences). Monocytes were isolated from bone marrow using the EasySep mouse monocyte enrichment kit (STEMCELL Technologies, 19861). Live DC1 (CD11c^+^ MHC class II^+^ XCR1^+^) and DC2 (CD11c^+^ MHC Class II^+^ Sirpa^+^) and NK (NK1.1^+^) cells were bulk sorted from spleens using a BD FACS Aria flow cytometer. Pan T cells were isolated from spleens using the EasySep Mouse T Cell Isolation Kit (STEMCELL Technologies, 19851).

### In vitro cell killing assay.

MC38, B16F10, KPC, and Pan02 cells expressing luciferase were used as target cells. For neutrophil-mediated cell killing assay, target cells were cocultured for 24–48 hours with neutrophils at various E:T ratios (e.g., 20:1) in presence or absence of indicated stimulant. In ROS inhibition experiments, neutrophils were pretreated with 300 μM Apocynin (Tocris Biosciences) or 5 μM diphenyleneiodonium chloride (Tocris Biosciences) and then used for target cell killing. For NK cell and neutrophil combination experiments, target cells were cultured at a E:T ratio of 10:1 NK cells and 2:1 neutrophils or together for 48 hours. In experiments, where specified, supernatants from either control- or IL-36–treated neutrophils were added instead of neutrophils. Luciferase was assessed using Steadyglo (Promega). and luminescence was read using a luminometer (Envision, PerkinElmer).

### ROS measurement.

ROS was measured as described before (49). Briefly, neutrophils pretreated with rmIL-36γ overnight were treated with 1 μg/mL LPS (Invivogen) or irradiated tumor cells for 30 minutes at 37°C in the presence of 5 mM CM-H2DCFDA (Invitrogen) to measure total intracellular ROS. Cells were washed with PBS and analyzed by FACS to examine mean florescence intensity of CM-H_2_DCFDA.

### Cytokine measurement.

Supernatants of neutrophils cultured in the presence or absence of rmIL-36γ were analyzed using the V-PLEX Mouse cytokine 19-Plex MSD kit (Mesoscale Discovery, K15255D) following the manufacturer’s instructions.

### ELISPOT assay.

Total T cells were isolated from spleens of MC38-bearing animals. Neutrophils were isolated from bone marrow of WT or IL-36R–KO animals and treated with rmIL-36γ for 2 hours. MC38 cell lysate was prepared by irradiating the cell line at 750 Gy (CellRad) at 4 × 10^6^ per mL, and lysate of 2.5 × 10^4^ MC38 cells was added to rmIL-36γ–treated neutrophils for 2 hours. Total T cells (2 × 10^5^) and neutrophils (10^5^) were added to the wells of ELISPOT plates precoated with a murine IFN-γ capture antibody. Alternatively, T cells and neutrophils were cultured with H-2K^b^ binding Adpgk (ASMTNMELM) peptide in the presence or absence of rmIL-36γ. For B16F10 experiments, splenocytes isolated from tumor-bearing mice were incubated with indicated neoantigen peptides. Samples were cocultured for 18 hours at 37°C with 5% CO^2^ incubator. IFN-γ^+^ spots on ELISPOT plates were developed according to the manufacturer’s instructions (BD Biosciences, 551083).

### T cell proliferation assay.

Splenic pan–T cells were labeled with 2.5–5 μM Cell Trace Violet Proliferation dye (Thermo Fisher Scientific, C34571) at 37°C for 20 minutes. Labeling was stopped by adding 10× excess complete RPMI media containing 10% FBS and incubated for 5 minutes at room temperature before pelleting the cells and resuspending in complete RPMI. 0.5 × 10^5^ to 1 × 10^5^ cells/well were added in duplicates or triplicates to a flat-bottom 96-well plate precoated with 2 μg/mL anti-CD3 (Biolegend, 100340). Purified bone marrow neutrophils were added at a 1:1 ratio. Soluble anti-CD28 (BD Biosciences, 553295) was added at 1 μg/mL with 5 ng/mL IL-2 (Peprotech, 212-12). Recombinant mIL-36γ was added at 500 ng/mL if indicated. T cells and neutrophils were cocultured either alone or in the presence of irradiated tumor cells (10^5^/well), 1 μM CpG Class C (Invivogen, tlrl-2395), or 1 μg/mL R848 (Invivogen, tlrl-r848). T cell proliferation was measured after 72 hours using flow cytometry. For IL-36γ measurement in supernatants, BaF/3 luciferase reporter cells were cultured with the supernatant, and luciferase signal was assayed. rmIL-36γ was used to generate a standard curve, and amounts of IL-36γ were extrapolated using the standard curve.

For OT1 proliferation assay, 100,000 Cell Trace Violet–labeled OT1 cells and 100,000 bone marrow neutrophils were cultured in the presence of rmIL-36γ or cocultured with OVA protein at 100 μg/mL in the presence or absence of rm IL-36γ for 2 days. ConA (eBioscience, 00-4978-03) was used as positive control, and cells were analyzed by flow cytometry.

### OVA protein uptake assay.

OVA protein was labeled with Alexa Fluor 647 (ThermoFisher Scientific, SA20186). Neutrophils were incubated with or without 500 ng/mL rmIL-36γ for 2 days. 100,000 neutrophils were pulsed with 100 μg/mL Alexa Fluor 647–labeled OVA for 1 hour at 37°C with 5% CO_2_ in an incubator. Cells were washed and stained for analysis by flow cytometry.

### Western blot.

Western blot analysis was performed as described before (49). Neutrophils were lysed in solution containing 10% glycerol, 3% SDS, 1 mM PMSF, and 5 mM NaF and heated immediately at 95°C for 5 minutes. Equal amounts of proteins, measured using BCA assay (Pierce), were separated by SDS-PAGE on 4%–12% Bis-Tris gels (Invitrogen) and transferred onto nitrocellulose membranes using iBlot (Invitrogen). Membranes were blocked for 1 hour at room temperature with 5% milk in TBST and probed with indicated antibody in 5% milk overnight at 4°C. Primary antibodies (Cell Signaling Technology) included anti-phosphop38 (catalog 4511), anti-p38 (catalog 8690), anti-phospho-Ikbalpha (catalog 9246), anti-Ikbalpha (catalog 4814), anti-phospho-p65 (catalog 3033), anti-p65 (catalog 8242), and HSP90 (catalog 4877). HRP-conjugated anti-rabbit (Cell Signaling Technology, 7074), anti-mouse (Cell Signaling Technology, 7076), and anti–Armenian hamster (Jackson Immunoresearch, 127-035-160) secondary antibodies were used. Image quantification was performed using ImageJ software (NIH).

### Bulk RNA-Seq sample and library preparation.

Neutrophils, NK cells, monocytes, and DCs were isolated from bone marrow or spleens as described above. Bone marrow–derived macrophages (BMDMs) were generated by culturing isolated bone marrow cells with 50 ng/mL M-CSF (R&D Technologies) for 7 days. Each cell type was either untreated or treated with 500 ng/mL rmIL-36γ (R&D Technologies) for 6 or 24 hours, following which RNA was extracted using the RNeasy isolation kit (Qiagen). Depending on the RNA amount available, 0.7– 5.0 ng total RNA was used as the starting input to generate the full-length cDNA by using the SMART-Seq v4 Ultra-low Input RNA Kit for Sequencing (Takara, 634890). Preparation of SMARTer cDNA was sequentially amplified by following the LD-PCR procedure, such that the thermal cyclic program was according to the input amount of total RNA: 11 PCR cycles for 1.0–5.0 ng RNA samples and 15 PCR cycles for 0.7–1.0 ng RNA samples. The LD-PCR amplified cDNA was purified and further validated in the Agilent High Sensitivity D5000 ScreenTape (Agilent, 5067-5592) on the Agilent 4200 TapeStation. Then, 300 pg cDNA products were used for library preparation in each reaction by using the Illumina Nextera XT kit and following the manual’s instructions (Illumina, FC-131-1096). The products of low-input cDNA libraries were then barcoded during 12 cycles of thermal program with specific index adapters for Illumina sequencing (Nextera XT Index Set D, FC-131-2004). The resulting libraries were cleaned up using 0.8× AMPure XP beads (Beckman Coulter, A63881) and eluted in 25 μL resuspension buffer. After library QC and quantitation, libraries were sequenced to a minimum depth of 45 million paired-end/dual-indexed 2 × 150 bp reads on an Illumina HiSeq4000. RNA-Seq reads (Illumina HiSeq platform, 75 bp paired-end sequencing) were aligned to human genome build 38, and fragments per kilobase per million sequenced, quantile normalized (FPKQ) values were determined using Array Suite software (Omicsoft) and in-house software.

### Bulk RNA-Seq data analysis.

Salmon v1.4.0 was used with default parameters to quasi-map RNA-Seq reads to the target transcriptome (Gencode vM27) for quantification. The count files were then imported into DESeq2 (v1.34.0 in R4.1) using tximport and DESeqDataSetFromTximport (50). A minimal prefiltering cutoff of 10 reads across samples for each gene was done to remove lowly expressed genes. Differential expression analysis was done using DESeq2 default parameters comparing IL-36–treated to the untreated sample for each cell type, unless specified otherwise. Genes with greater than 2-fold difference and adjusted *P* values of less than 0.05 were considered differentially expressed. Gene set enrichment analysis (GSEA) was done using the standalone software (v3.1) with differentially expressed genes analyzed with MsigDB Hallmark gene sets (47, 51, 52) and other custom gene sets as labeled on corresponding GSEA plots. Heatmaps were generated using Morpheus (Broad Institute).

### Survival analysis.

Survival analysis of TCGA data was done using GEPIA2 (53). Neutrophil signature was derived from a subset of the following genes: *Amica1*, *Anpep*, *Csf3r*, *Cxcr1*, *Cxcr2*, *Evi2b*, *Fcg3rb*, *Fpr1*, *Fpr2*, *Gos2*, *Mnda*, *Pde4b*, and *Tlr8*. IL-36 signature was created using a subset of the following genes: *Il36a*, *Il36b*, and *Il36g*. For both signatures, the most prognostic genes were identified using the survival map feature in GEPIA2. For HNSC and CESC, *Fpr1* and *Cxcr1* were found to be the prognostic neutrophil markers, and *Il36a* and *Il36a*/*Il36b* were used for IL-36 signature genes, respectively. We combined these genes using a median quartile score and assessed overall survival using subsets of patients with high (75 percentile and higher) versus low (25 percentile and lower) signatures.

### Statistics.

Statistical significance was calculated by Prism 8 software (Graphpad). Tumor volumes in mouse in vivo studies were compared using a repeated measures 2-way ANOVA followed by Šidák’s (2 groups) or Tukey’s (>2 groups) multiple comparisons. For pairwise comparison, we used unpaired 2-tailed Mann-Whitney *t* test for 2-group comparisons or 1-way ANOVA followed by Tukey’s multiple comparison test for 3 or more groups. Values of *P* < 0.05 were considered significant.

### Study approval.

All animal experimental protocols were approved by the Institutional Animal Care and Use Committee of Amgen and were conducted in accordance with the guidelines set by the Association for Assessment and Accreditation of Laboratory Animal Care.

### Data availability.

The normalized read counts and differentially expressed genes from bulk RNA-Seq data of IL-36–mediated gene expression changes in diverse innate cells, including neutrophils, DC1s, DC2s, NK cells, BMDMs, and monocytes are included in Supplemental Tables 1 and 2, and the raw data were uploaded in NCBI’s Gene Expression Omnibus database (GEO GSE210120).

## Author contributions

SR, KF, AL, CWL, AK, JK, PO, AM, XL, and RN conducted experiments. AR, H Zhao, H Zhou, and HX generated critical reagents. HM, CML, BVL, ST, CET, JD, JE, and LCW provided resources and guidance. SR and RN wrote manuscript. RN conceptualized and supervised the work.

## Supplementary Material

Supplemental data

Supplemental table 1

Supplemental table 2

Supplemental table 3

## Version 1 06/15/2023

Electronic publication
