# Supplementary material for: Autonomous IL-36R signaling in neutrophils activates potent antitumor effector functions
Source: J Clin Invest. 2023 Jun 15;133(12):e162088. doi: 10.1172/JCI162088 (PMC10266786; doi:10.1172/JCI162088)
Supplement: Supplemental data [file jci-133-162088-s172.pdf]

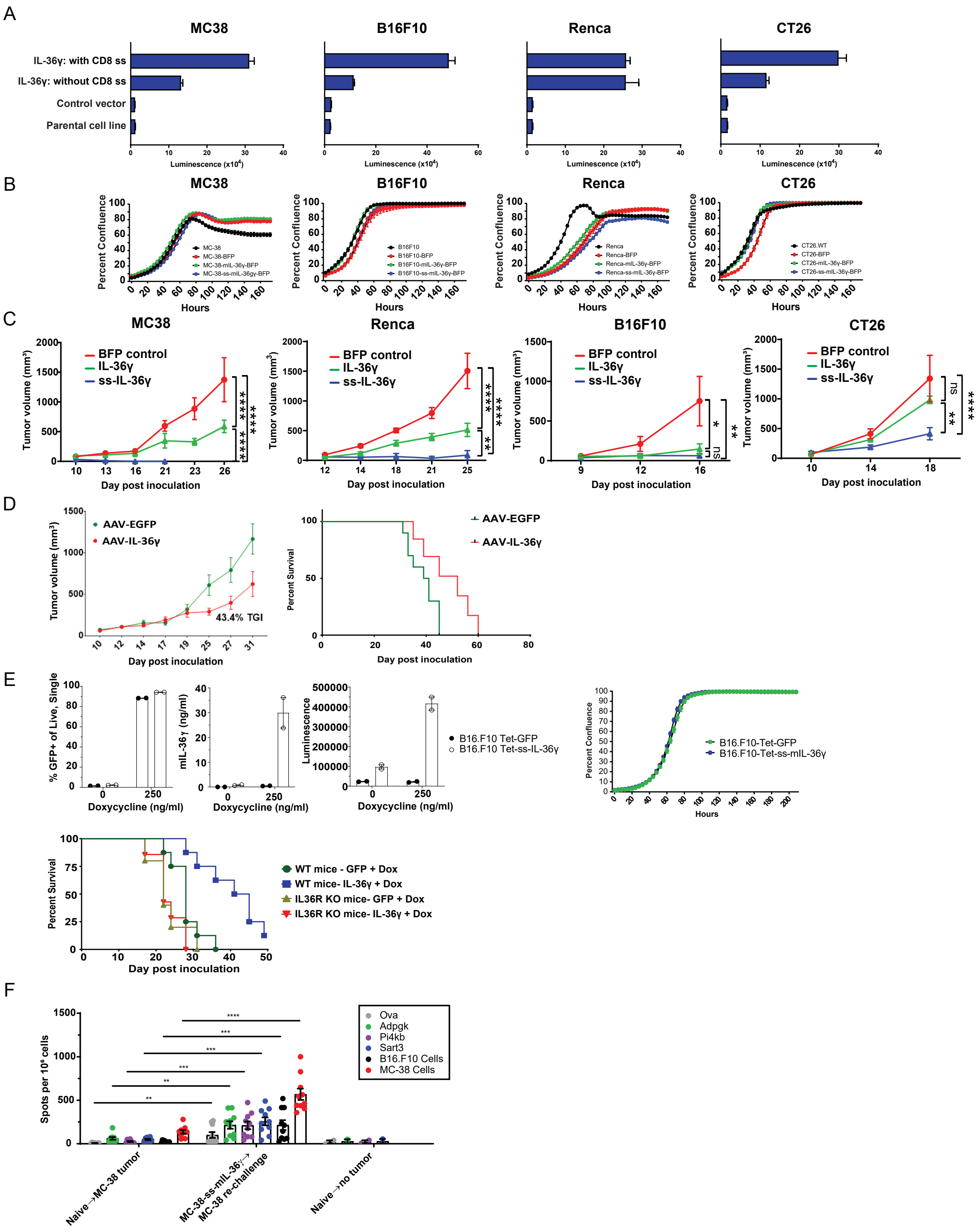
A
MC38
B16F10
Renca
CT26
IL-36γ: with CD8 ss
IL-36γ: without CD8 ss
Control vector
Parental cell line
Luminescence (x10^4)
B
MC38
B16F10
Renca
CT26
Percent Confluence
Hours
MC-38
MC-38-BFP
MC-38-mIL-36γ-BFP
MC-38-ss-mIL-36gγ-BFP
B16F10
B16F10-BFP
B16F10-mIL-36γ-BFP
B16F10-ss-mIL-36γ-BFP
Renca
Renca-BFP
Renca-mIL-36γ-BFP
Renca-ss-mIL-36γ-BFP
CT26.WT
CT26-BFP
CT26-mIL-36γ-BFP
CT26-ss-mIL-36γ-BFP
C
MC38
Renca
B16F10
CT26
BFP control
IL-36γ
ss-IL-36γ
Tumor volume (mm³)
Day post inoculation
D
AAV-EGFP
AAV-IL-36γ
Tumor volume (mm³)
43.4% TGI
Day post inoculation
Percent Survival
E
% GFP+ of Live, Single
mIL-36γ (ng/ml)
Luminescence
Doxycycline (ng/ml)
B16.F10 Tet-GFP
B16.F10 Tet-ss-IL-36γ
Percent Confluence
B16.F10-Tet-GFP
B16.F10-Tet-ss-mIL-36γ
Hours
Percent Survival
WT mice - GFP + Dox
WT mice- IL-36γ + Dox
IL36R KO mice- GFP + Dox
IL36R KO mice- IL-36γ + Dox
Day post inoculation
F
Spots per 10⁶ cells
Ova
Adpgk
Pi4kb
Sart3
B16.F10 Cells
MC-38 Cells
Naive→MC-38 tumor
MC-38-ss-mIL-36γ→
MC-38 re-challenge
Naive→no tumor

Supplementary Figure 1. IL-36 exhibits potent anti-tumor immune response via effects on hematopoietic cells.

(A) Bioactivity of secreted IL-36γ in the supernatant of indicated syngeneic cell line was assessed by luciferase assay using BaF/3 cells engineered to overexpress murine IL-36R and NFkB luciferase under the control of IL-8. Luminescence readings after 24h of cell cultures are shown. ss = signal sequence. (B) In vitro growth of indicated cell line assessed using Incucyte over a period of one week. (C) Tumor growth of control or IL-36γ expressing MC38, B16F10, Renca and CT26 syngeneic tumor models. Tumor growth inhibition (TGI) curves are shown. ssIL-36 = signal sequence-IL36. (D) Tumor growth of MC38 tumors treated with AAVs expressing GFP or IL-36γ starting on day 10. Tumor growth inhibition (TGI) (left) and survival plot (right) is shown. n=10/group. (E) Characterization of doxycycline (Dox) inducible expression of IL-36γ. Induction of GFP (top left bar graph), amount of IL-36γ (middle bar graph) and bioactivity of secreted IL-36γ (right bar graph) upon dox treatment. In vitro growth curve using Incucyte (top) and in vivo survival curves (bottom) are shown. (F) MC38 cells implanted into naïve WT mice or mice that rejected primary tumor expressing IL-36γ. IFNγ ELISPOT assay (right) using splenocytes from tumor-bearing mice pulsed with control (SIINFEKL peptide) or neoantigen peptides (Adpgk, Pi4kb, or Sart3) or B16F10 cells or MC38 cells. Number of IFNγ-positive spots after 24h is shown. Data are shown as mean±SEM, Two-Way ANOVA followed by Tukey's multiple comparison test (C, E), one-way ANOVA followed by Tukey's multiple comparison (F). *$p<0.05$, **$p<0.01$, ***$p<0.001$, ****$p<0.0001$. ns, not significant. ss= signal sequence. Data is representative of two (A, B, D, F) and three (C) independent experiments.

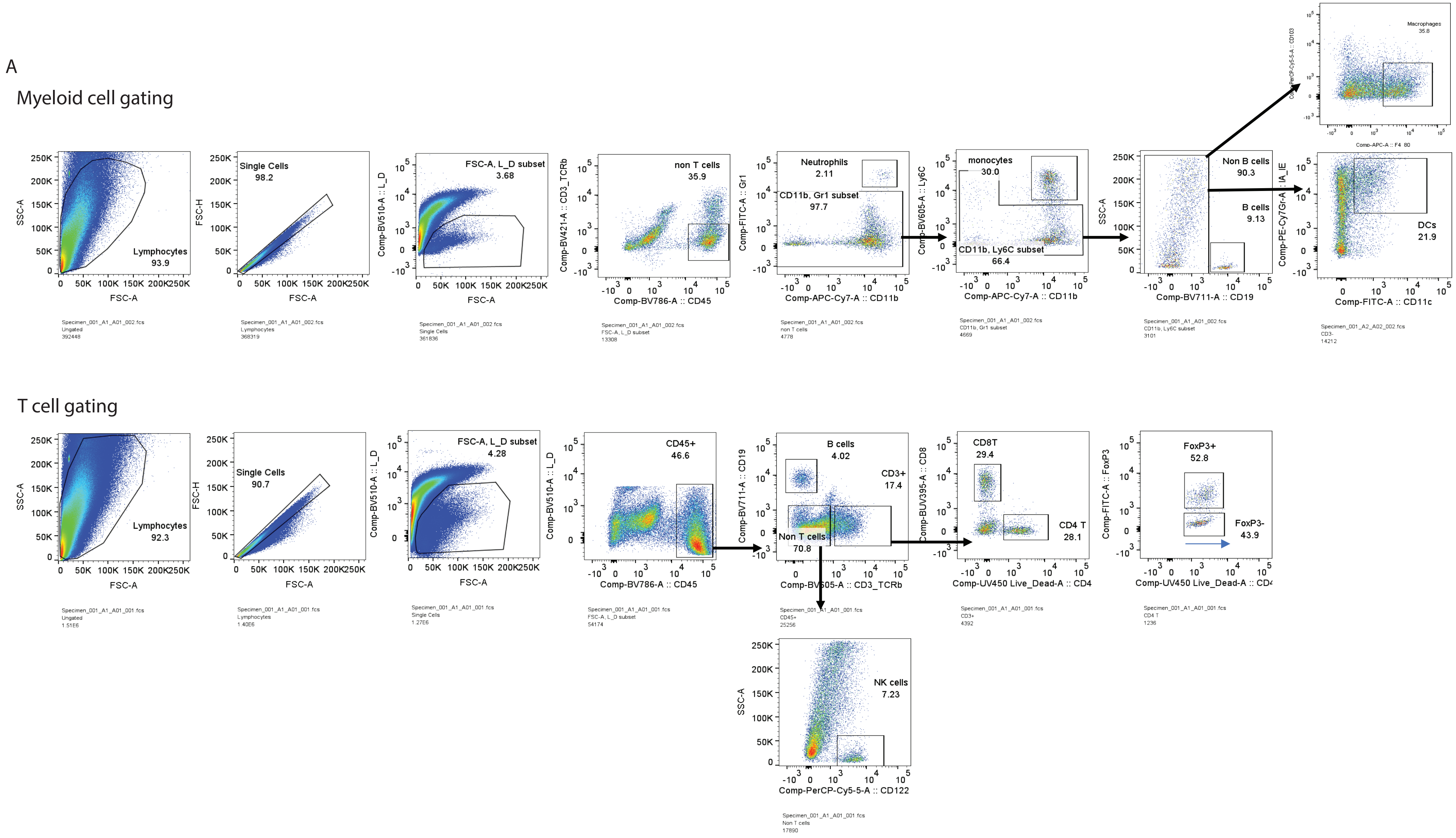
A
Myeloid cell gating
Lymphocytes 93.9
Single Cells 98.2
FSC-A, L_D subset 3.68
non T cells 35.9
Neutrophils 2.11
CD11b, Gr1 subset 97.7
monocytes 30.0
CD11b, Ly6C subset 66.4
Non B cells 90.3
B cells 9.13
DCs 21.9
Macrophages 35.8
T cell gating
Lymphocytes 92.3
Single Cells 90.7
FSC-A, L_D subset 4.28
CD45+ 46.6
B cells 4.02
CD3+ 17.4
Non T cells 70.8
NK cells 7.23
CD8T 29.4
CD4 T 28.1
FoxP3+ 52.8
FoxP3- 43.9
SSC-A
FSC-A
FSC-H
Comp-BV510-A :: L_D
Comp-BV421-A :: CD3_TCRb
Comp-BV786-A :: CD45
Comp-FITC-A :: Gr1
Comp-APC-Cy7-A :: CD11b
Comp-BV605-A :: Ly6C
Comp-BV711-A :: CD19
Comp-PE-Cy7Gr-A :: IA_IE
Comp-FITC-A :: CD11c
Comp-APC-A :: F4 80
Comp-BV605-A :: CD3_TCRb
Comp-PerCP-Cy5-5-A :: CD122
Comp-BUV395-A :: CD8
Comp-UV450 Live_Dead-A :: CD4
Comp-FITC-A :: FoxP3

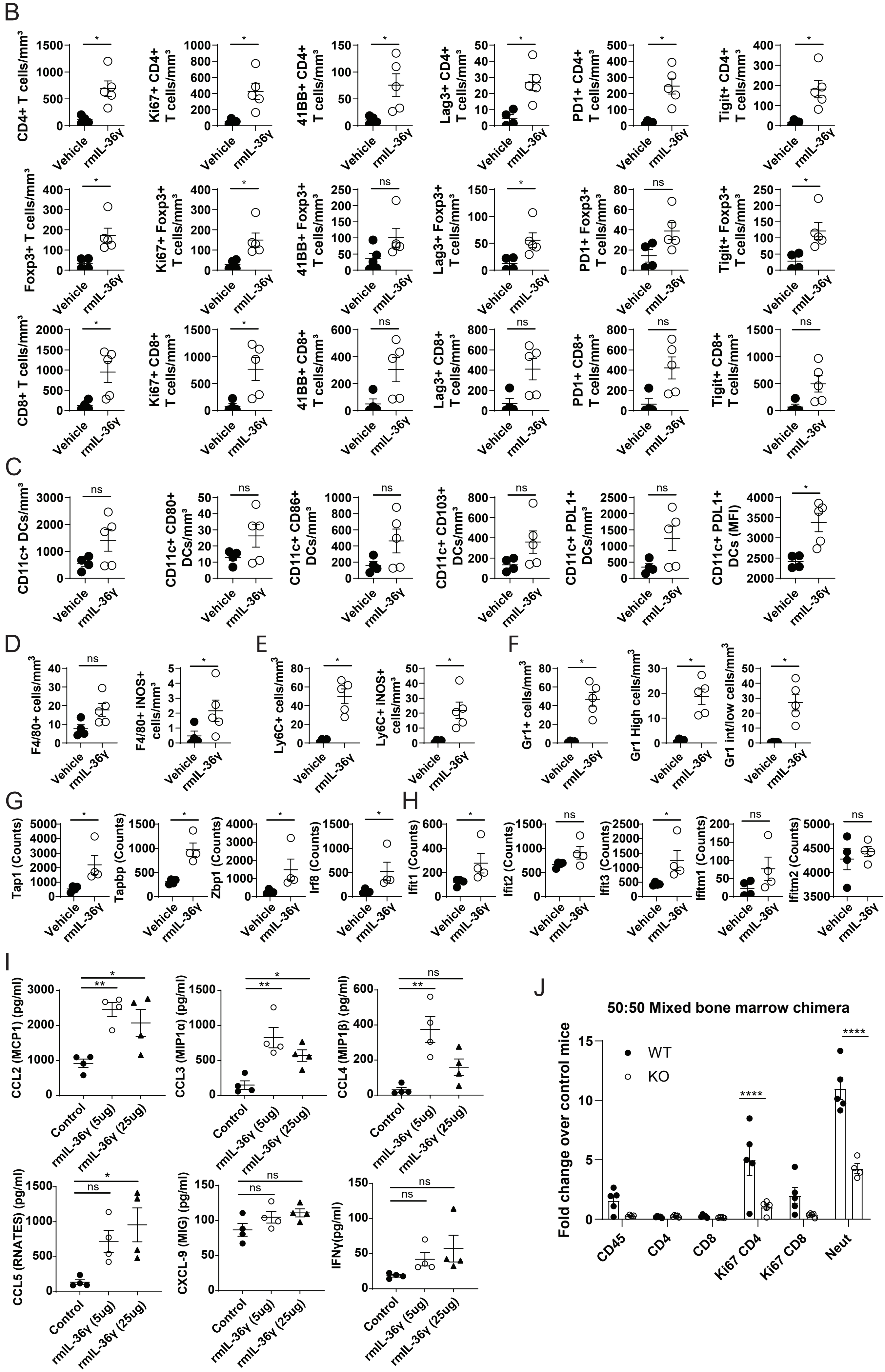
B
CD4+ T cells/mm³
Ki67+ CD4+ T cells/mm³
41BB+ CD4+ T cells/mm³
Lag3+ CD4+ T cells/mm³
PD1+ CD4+ T cells/mm³
Tigit+ CD4+ T cells/mm³
Foxp3+ T cells/mm³
Ki67+ Foxp3+ T cells/mm³
41BB+ Foxp3+ T cells/mm³
Lag3+ Foxp3+ T cells/mm³
PD1+ Foxp3+ T cells/mm³
Tigit+ Foxp3+ T cells/mm³
CD8+ T cells/mm³
Ki67+ CD8+ T cells/mm³
41BB+ CD8+ T cells/mm³
Lag3+ CD8+ T cells/mm³
PD1+ CD8+ T cells/mm³
Tigit+ CD8+ T cells/mm³
C
CD11c+ DCs/mm³
CD11c+ CD80+ DCs/mm³
CD11c+ CD86+ DCs/mm³
CD11c+ CD103+ DCs/mm³
CD11c+ PDL1+ DCs/mm³
CD11c+ PDL1+ DCs (MFI)
D
F4/80+ cells/mm³
F4/80+ iNOS+ cells/mm³
E
Ly6C+ cells/mm³
Ly6C+ iNOS+ cells/mm³
F
Gr1+ cells/mm³
Gr1 High cells/mm³
Gr1 int/low cells/mm³
G
Tap1 (Counts)
Tapbp (Counts)
Zbp1 (Counts)
Irf8 (Counts)
H
Ifit1 (Counts)
Ifit2 (Counts)
Ifit3 (Counts)
Ifitm1 (Counts)
Ifitm2 (Counts)
Vehicle
rmIL-36γ
I
CCL2 (MCP1) (pg/ml)
CCL3 (MIP1α) (pg/ml)
CCL4 (MIP1β) (pg/ml)
CCL5 (RNATES) (pg/ml)
CXCL-9 (MIG) (pg/ml)
IFNγ(pg/ml)
Control
rmIL-36γ (5ug)
rmIL-36γ (25ug)
J
50:50 Mixed bone marrow chimera
Fold change over control mice
WT
KO
CD45
CD4
CD8
Ki67 CD4
Ki67 CD8
Neut

Supplementary Figure 2. Enhanced IL-36 signaling within TME impacts both innate and adaptive immune cells.

(A-F) FACS analysis of immune cells in the TME of control B16F10 tumors (n=5) or those with intratumoral administration of 25 ug/kg of rmIL-36γ (n=5). (A) shows gating strategy (B) CD4 T cells (top), Treg cells (middle) and CD8 T cells (bottom), (C) Dendritic cells, (D) F4/80+ Macrophages, (E) Ly6C+ monocytes, (F) Gr1+ neutrophils. Numbers were normalized to the tumor volume at harvest. (G-H) Nanostring analysis of control or rmIL-36γ treated B16F10 tumors showing select genes involved in (G) antigen presentation and processing (H) type I Interferon signature. Y-axis represents transcript counts of individual genes. (I) CBA analysis showing various proinflammatory chemokines and cytokines in the tumor lysate of control B16F0 tumors or with intratumoral injection of rmIL-36γ. (J) Fold increase in the number of various immune cells in bone marrow chimera mice harboring either WT or IL-36R-deficient (KO) hematopoietic cells and implanted with B16F10. Mice were treated with 25ug/kg rmIL-36γ. Fold increase was calculated by dividing average of individual cell type in rmIL-36γ treated mice with the corresponding control mice. Data are shown as mean±SEM, Unpaired two-tailed Mann-Whitney t-test (B-H, J), One-way ANOVA followed by Tukey's multiple comparison (I). *$p<0.05$, **$p<0.01$, ****$p<0.0001$. ns, not significant. Data is representative of two independent experiments.

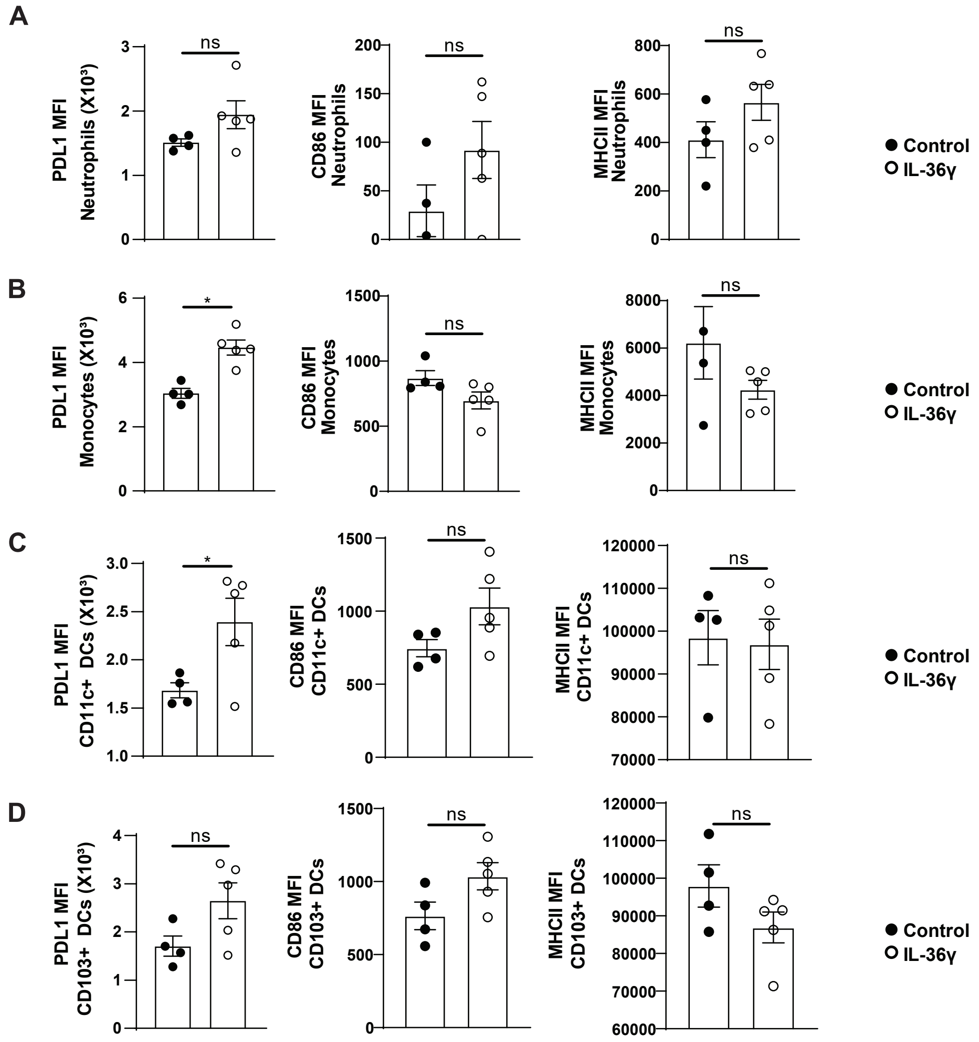

Supplementary Figure 3. IL-36 regulates tumor growth in the absence of T and B cells. (A-F) FACS analysis showing indicated activation marker for various innate immune cells in the TME of control (n=5) or IL-36γ-expressing MC38 tumors (n=5) in RagKO mice. (A) neutrophils (B) monocytes (C) CD11c+ DCs and (D) CD103+ DCs. Data are shown as mean ±SEM, Unpaired two-tailed Mann-Whitney t-test, *p<0.05. ns, not significant. Data is representative of two independent experiments.

A

Population	Tissue	Isolation method	Cell culture media	IL-36g concentration (treatment group)	Time point(s) collected
Neutrophils	Bone Marrow	Neutrophil enrichment kit	cRPMI	500 ng/mL	6 hr, 24 hr
Monocytes	Bone Marrow	Monocyte isolation kit			
BMDM	Bone Marrow	Differentiated for 7 days from isolated monocytes, using 100ng/mL rmM-CSF	cDMEM		
NK	Spleen	Sort	cRPMI (- P/S)		
DC1	Spleen	Sort	cIMDM + 100ng/ml Flt3L		
DC2	Spleen	Sort	cIMDM + 100ng/ml Flt3L		

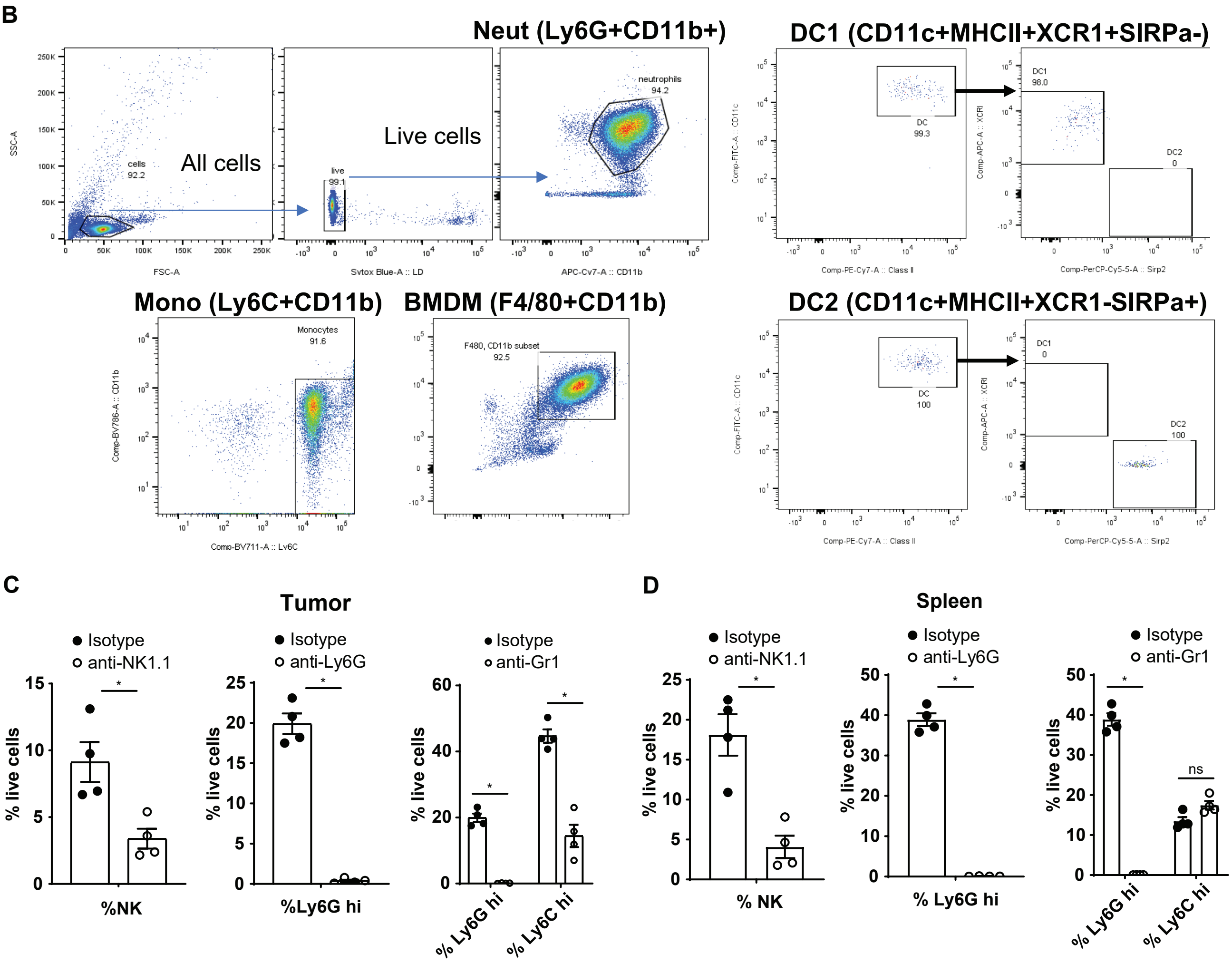

Supplementary Figure 4. Neutrophils and NK cells contribute to IL-36-mediated anti-tumor responses in RagKO mice.

(A) Table showing the source and strategy of bulk RNAseq of various innate immune cells (B) Gating strategy and purity of various innate immune cells used in bulk RNAseq experiment. Markers used for purification of the cells is shown. (C-D) FACS analysis showing frequency of indicated immune cells in (C) tumor (D) spleen upon treatment with depleting monoclonal antibodies (mAb) in RagKO mice harboring control or IL-36γ expressing MC38 tumors. Anti-NK1.1. mAb for depleting NK cells, Ly6G mAb to deplete neutrophils and Gr-1 mAb to deplete both neutrophils and monocytes. Data are shown as mean±SEM, Unpaired two-tailed Mann-Whitney t-test, *$p<0.05$. ns, not significant. Data is representative of two independent experiments (C,D). For (A,B) cells were purified in three independent experiments for each type, in each experiment two technical replicates were used for each condition and cell type. After RNA extraction and before sequencing technical replicates were combined and treated as one sample.

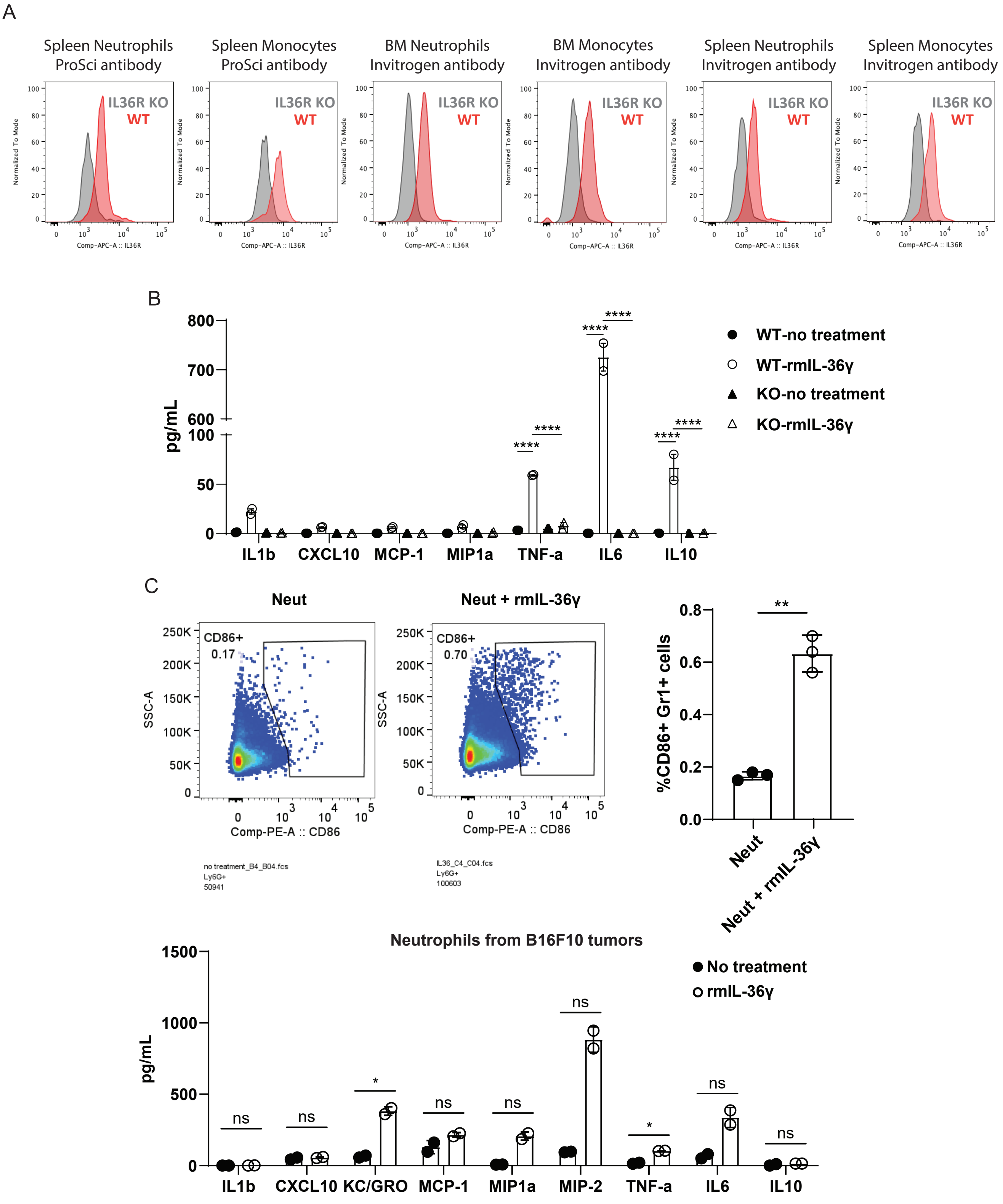
A
Spleen Neutrophils ProSci antibody
Spleen Monocytes ProSci antibody
BM Neutrophils Invitrogen antibody
BM Monocytes Invitrogen antibody
Spleen Neutrophils Invitrogen antibody
Spleen Monocytes Invitrogen antibody
IL36R KO
WT
Normalized To Mode
Comp-APC-A :: IL36R
B
pg/mL
WT-no treatment
WT-rmIL-36γ
KO-no treatment
KO-rmIL-36γ
****
IL1b
CXCL10
MCP-1
MIP1a
TNF-a
IL6
IL10
C
Neut
Neut + rmIL-36γ
CD86+
0.17
0.70
SSC-A
Comp-PE-A :: CD86
no treatment_B4_B04.fcs
Ly6G+
50941
IL36_C4_C04.fcs
Ly6G+
100603
%CD86+ Gr1+ cells
**
Neutrophils from B16F10 tumors
No treatment
rmIL-36γ
pg/mL
ns
*
IL1b
CXCL10
KC/GRO
MCP-1
MIP1a
MIP-2
TNF-a
IL6
IL10

Supplementary Figure 5. Cell autonomous IL-36 signaling is a potent activator of neutrophils. (A) IL-36R expression analysis on neutrophils and monocytes from bone marrow and spleen by flow cytometry using anti-IL-36R Ab (Invitrogen [PA5] or Prosci [7501]). Cells were gated on CD11b+Ly6G+ for neutrophils and CD11b+Ly6C+ for monocytes. Histograms for WT and IL-36R KO (KO) are shown. (B) Production of proinflammatory cytokines and chemokines by neutrophils isolated from bone marrow of WT or IL-36R-deficient (KO) mice and treated with 500 ng/ml of rmIL-36γ for 24h. Cytokines and chemokines were detected using multiplexed MSD kit. (C) IL-36 mediated activation of neutrophils as depicted by upregulation of CD86 with rmIL-36γ. Representative graphs and average of CD86-positive cells is shown in the bar graphs. (D) Production of proinflammatory cytokines and chemokines by neutrophils isolated from B16F10 tumors by magnetic enrichment using anti-Ly6G antibodies and treated with 500 ng/ml of rmIL-36γ for 24h. Cytokines and chemokines were detected using multiplexed MSD kit. Data are shown as mean±SEM, One-way ANOVA with Tukey's multiple comparison test (A), Unpaired two-tailed Mann-Whitney t-test (B,C). *$p<0.05$, **$p<0.01$, ****$p<0.0001$. ns, not significant. Data is representative of two independent experiments.

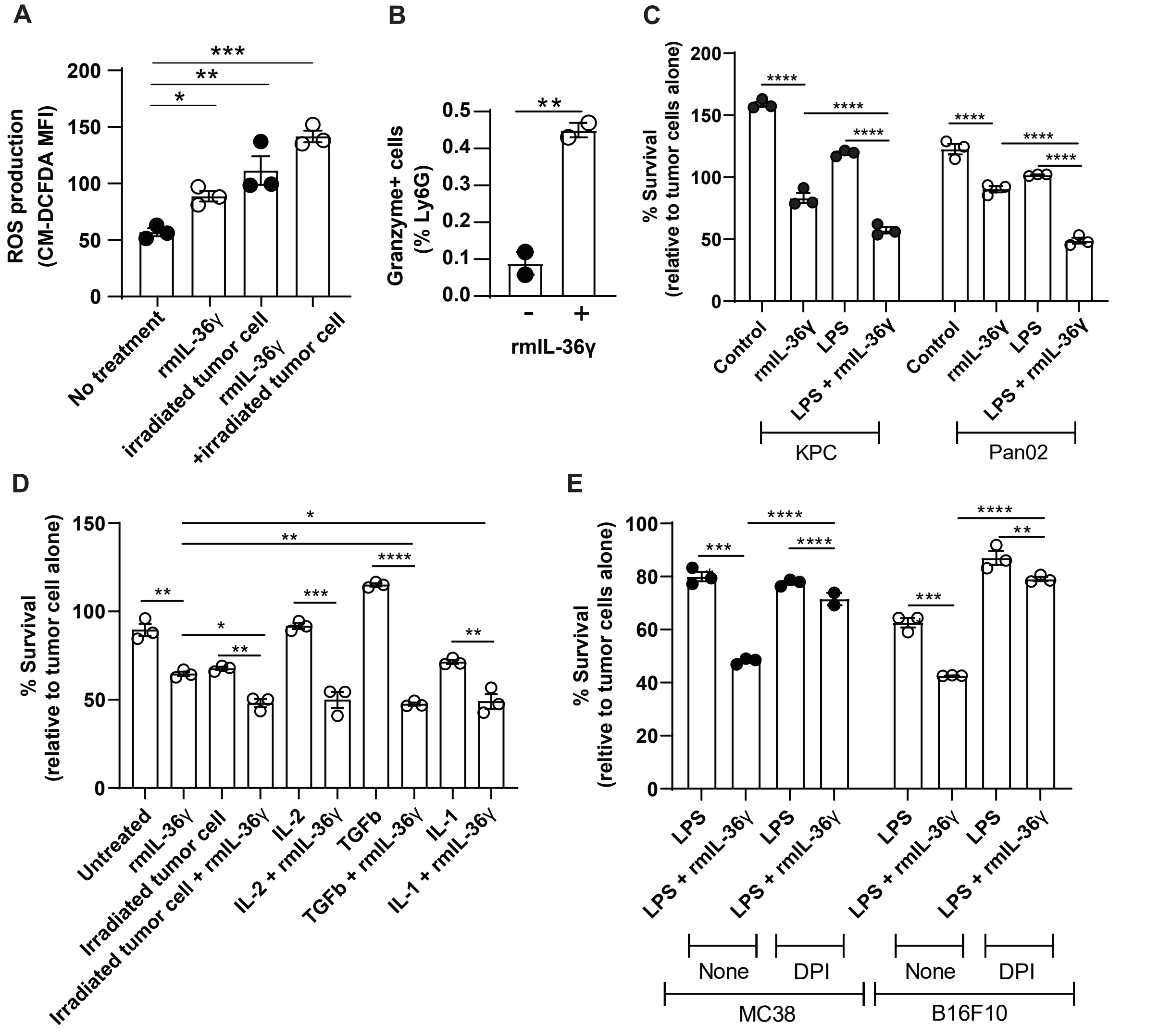

Supplementary Figure 6. IL-36 activated neutrophils directly kill tumor cells.
(A) ROS production by neutrophils either untreated or treated with 500 ng/ml of rmIL-36γ alone or in combination of irradiated MC38 tumor cells. ROS production as detected by increased MFI of ROS-sensitive dye CM-DCFDA. (B) Granzyme B production as detected by intracellular flow cytometry by neutrophils either untreated or treated with 500 ng/ml of rmIL- 36γ. (C) Direct killing of luciferase expressing KPC cells or Pan02 tumor cells by WT neutrophils either untreated or treated with indicated reagents, rmIL-36γ (500ng/ml), LPS (100ng/ml) for 48h. Data is represented as luciferase signal relative to target cells cultured without the presence of neutrophils. (D) Direct killing of MC38 cells expressing luciferase (MC38-Luc) by WT neutrophils either untreated or treated with indicated cytokine. Data is represented as luciferase signal relative to MC38-Luc cells cultured without neutrophils. (E) Direct killing of luciferase expressing MC38 cells or B16F10 tumor cells by WT neutrophils either untreated or treated with indicated reagents, rmIL-36γ (500ng/ml), LPS (100ng/ml) for 48h. For ROS inhibition, neutrophils were treated 5 uM of diphenyleneiodonium chloride (DPI) for 1h before adding to the culture. Data is represented as luciferase signal relative to target cells cultured without the presence of neutrophils. Data are shown as mean±SEM, One-way ANOVA with Tukey's multiple comparison test (A,C,D,E),Unpaired two-tailed Mann-Whitney t-test (B). *p<0.05, **p<0.01, ****p<0.001, ****p<0.0001. Data is representative of two (D,E) and three (A-C) independent experiments.

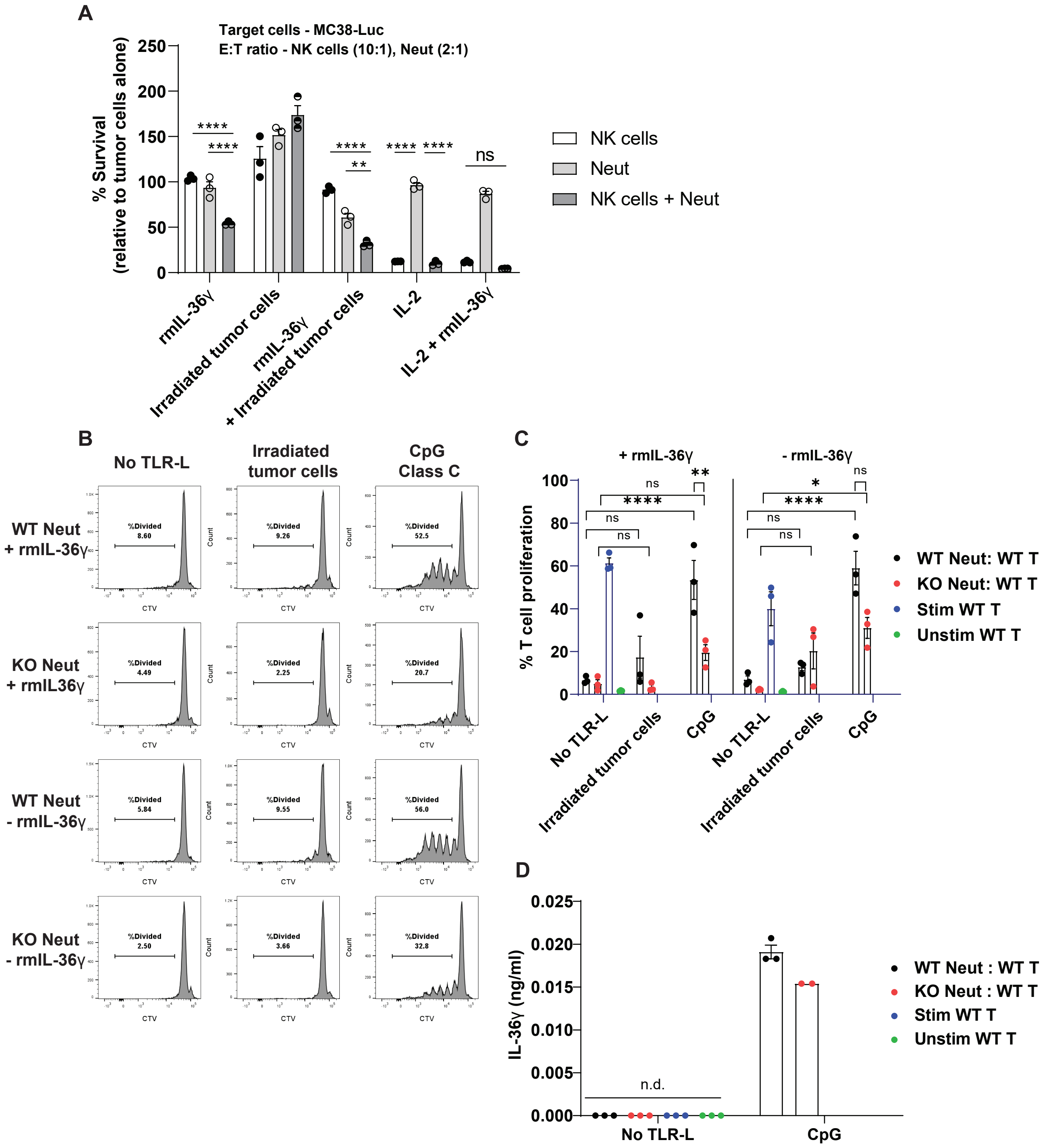

Supplementary Figure 7. IL-36 activated neutrophils modulate NK cell cytotoxicity and T cell proliferation.
(A) Cell killing of MC38 cells expressing luciferase (MC38-Luc) by NK cells alone, neutrophils alone or combination of NK cells and neutrophils. Cells were treated with indicated cytokine or irradiated tumor cell lysate. Data is represented as luciferase signal relative to MC38-Luc cells cultured without neutrophils. (B-D) Anti-CD3/CD28 stimulated proliferation of WT T cells cocultured with either WT neutrophils or IL-36R KO neutrophils for 72h in the presence (+) or absence (-) of 500 ng/ml of rmIL-36γ. Where indicated, 1 uM of CpG class C or MC38 tumor cell lysate was added. Representative histograms are shown in (B) and average proliferation of triplicate wells shown in (C). (D) IL-36γ present in the supernatant of cultures from no external rmIL-36γ added condition was assessed using BaF/3 reporter cells and amounts were extrapolated using rmIL-36γ standard curve. Data are shown as mean±SEM, two-way ANOVA with Tukey's multiple comparison's test. *p<0.05, ***p<0.001. ns, not significant. Data is representative of two independent experiments.

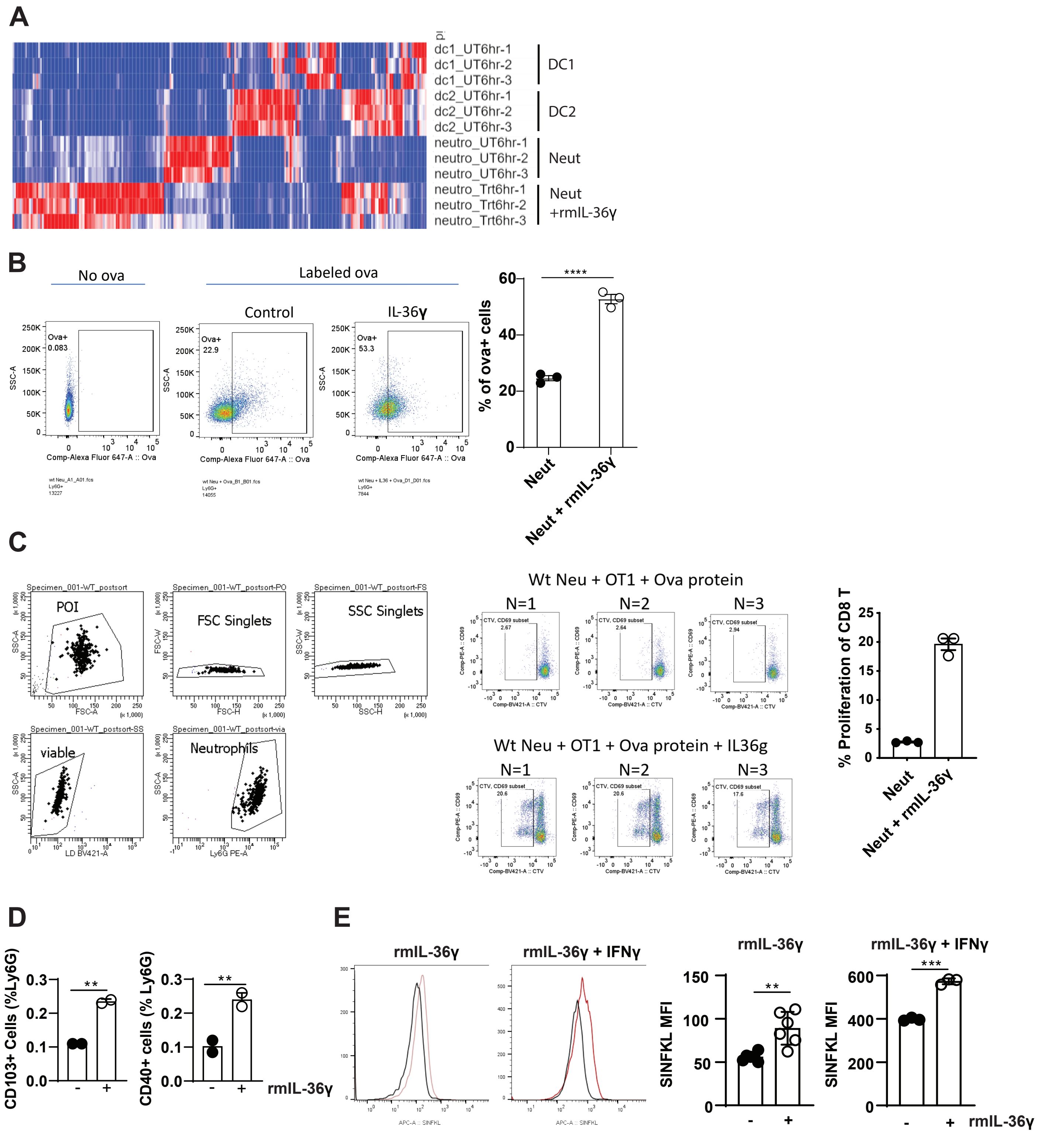

A
dc1_UT6hr-1
dc1_UT6hr-2
dc1_UT6hr-3
DC1
dc2_UT6hr-1
dc2_UT6hr-2
dc2_UT6hr-3
DC2
neutro_UT6hr-1
neutro_UT6hr-2
neutro_UT6hr-3
Neut
neutro_Trt6hr-1
neutro_Trt6hr-2
neutro_Trt6hr-3
Neut +rmIL-36γ
B
No ova
Labeled ova
Control
IL-36γ
Ova+ 0.083
Ova+ 22.9
Ova+ 53.3
SSC-A
Comp-Alexa Fluor 647-A :: Ova
% of ova+ cells
****
Neut
Neut + rmIL-36γ
C
POI
FSC Singlets
SSC Singlets
viable
Neutrophils
FSC-A
FSC-H
SSC-H
LD BV421-A
Ly6G PE-A
Wt Neu + OT1 + Ova protein
N=1
N=2
N=3
CTV, CD69 subset
2.67
2.64
2.94
Wt Neu + OT1 + Ova protein + IL36g
20.6
20.6
17.6
Comp-BV421-A :: CTV
Comp-PE-A :: CD69
% Proliferation of CD8 T
Neut
Neut + rmIL-36γ
D
CD103+ Cells (%Ly6G)
CD40+ cells (% Ly6G)
**
**
rmIL-36γ
E
rmIL-36γ
rmIL-36γ + IFNγ
APC-A :: SINFKL
SINFKL MFI
**
***
rmIL-36γ

Supplementary Figure 8. IL-36 activated neutrophils present antigen and modulate T cell proliferation.

(A) Heatmap showing differentially expressed genes of unstimulated DC1 and DC2 isolated from spleens of WT mice and neutrophils isolated from bone marrow, either untreated or treated with 500 ng/ml of rmIL-36γ for 6h. (B) WT neutrophils treated with rmIL-36γ for 48h were fed with florescent ova and its uptake was determined by FACS. Representative graphs (left) and average % positive cells is shown on the right. (C) Schematic for sorting neutrophils to 100% purity is shown on the left. OT-1 T cell proliferation for 48h in the presence of bone marrow neutrophils previously either untreated or treated with 500 ng/ml of rmIL-36γ for 2 h and then fed with ova protein. Representative graphs are shown in the middle panels and average of triplicates is shown in the bar graph on right. (D) Frequency of WT neutrophils positive for CD103 (left) and CD40 (right) with (+) or without (-) treatment with 500 ng/ml of rmIL-36γ for 24h. (E) Neutrophils from CAG-OVAL transgenic mice expressing ovalbumin under actin promoter were treated with rmIL-36γ alone (left) or in combination with 100 ng/ml of rmIFNγ (right) for 24h and SIINFEKL peptide presented on MHC class I was detected by FACS using anti- SIINFEKL antibody. Representative histograms are shown on the left and average MFI is shown on the right. (+) treated, (-) untreated. Data are shown as mean±SEM, Unpaired two-tailed Mann-Whitney t-test. **p<0.01, ***p<0.001, ****p<0.0001. Data is representative of two independent experiments.

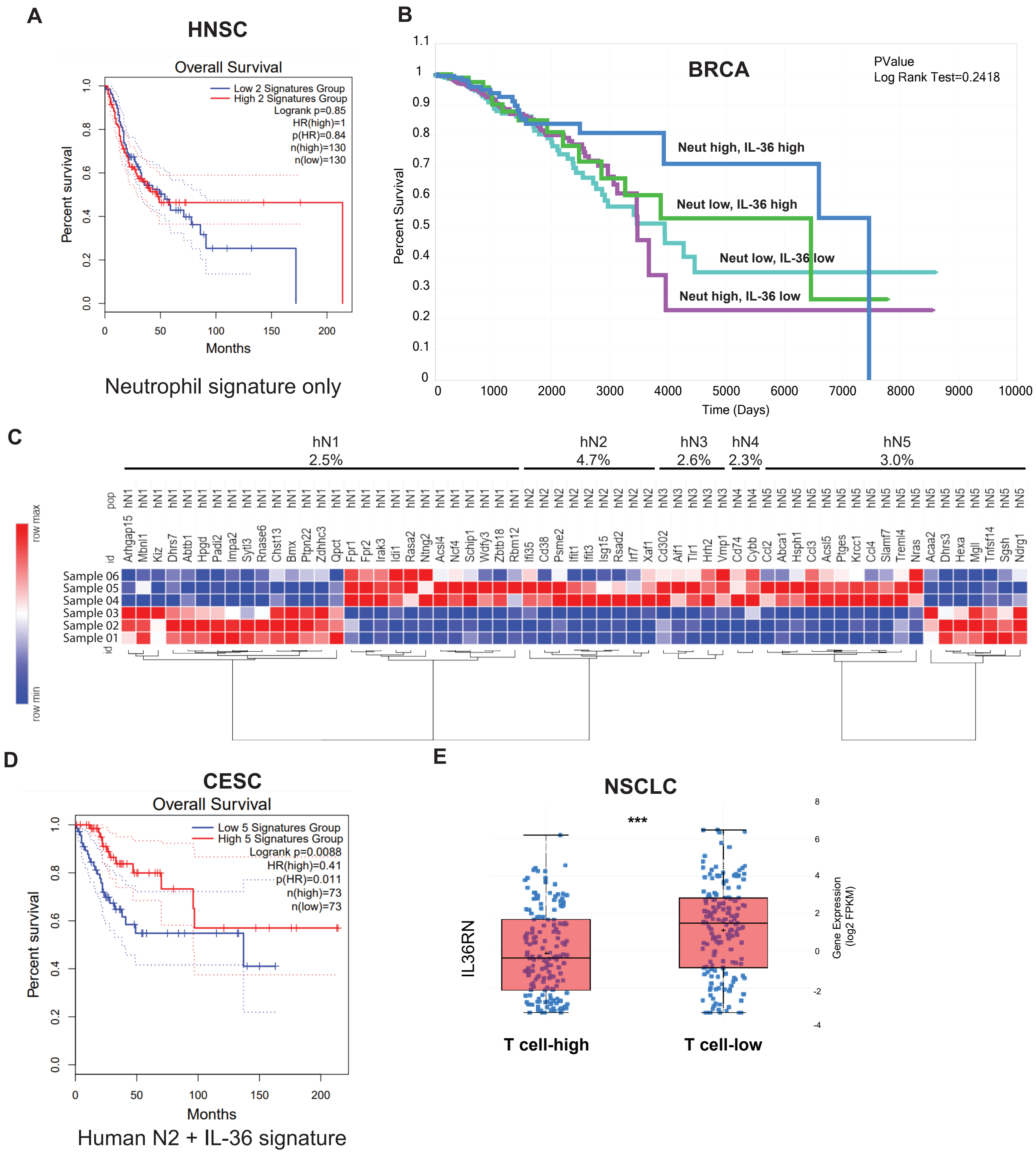

Supplementary Figure 9. Enhanced IL-36 signature combined with neutrophil signature predicts better survival in human cancer patients.

(A) aplan-Meier curve showing overall survival of patient subsets with high versus low Neutrophil signature in head and neck carcinoma (HNSC). (B) Kaplan-Meier curve showing overall survival of patient subsets with high versus low neutrophil and IL-36 signature in breast cancer (BRCA). Various combinations of neutrophil (N) and IL-36 are shown.

(C) Shared mouse and human neutrophil subset genes significantly enriched in IL-36γ treated neutrophils (500 ng/ml, 6hr). Enrichment (%) of IL-36γ induced gene signature in our dataset compared to literature dataset is shown on top of each subset. (D) Kaplan-Meier curve showing overall survival of patient subsets with high versus low neutrophil N2 and IL-36 signature in cervical squamous cell carcinoma and endocervical adenocarcinoma (CESC).

(E) TCGA analysis sh ng higher expression of IL-36RN associated with lower number of T cells in non-small lunc cancer (NSCLC).
