# Supplementary material for: Autonomous IL-36R signaling in neutrophils activates potent antitumor effector functions
Source: J Clin Invest. 2023 Jun 15;133(12):e162088. doi: 10.1172/JCI162088 (PMC10266786; doi:10.1172/JCI162088)
Supplement: Supplemental table 3 [file jci-133-162088-s175.docx]

Antibody lists

| Target | Conjugates | Clone | Cat# | Vendor | Experiment |
| --- | --- | --- | --- | --- | --- |
| CD8 | BUV395 | 53-6.7 | 563786 | BD Biosciences | PD profiling |
| CD4 | BUV496 | GK1.5 | 564661 | BD Biosciences |  |
| CD3 | BV605 | 2C11 | 563004 | BD Biosciences |  |
| TCRb | BV605 | H57-597 | 562840 | BD Biosciences |  |
| CD45 | BV785 | 30-F11 | 103149 | Biolegend |  |
| Ly6G | BUV395 | 1A8 | 563978 | BD Biosciences |  |
| Ly6C | BV605 | HK1.4 | 128036 | Biolegend |  |
| CD19 | BV711 | 6D5 | 115555 | Biolegend |  |
| CD11c | FITC | N418 | 117306 | Biolegend |  |
| CD11b | APC CY7 | M1/70 | 101227 | Biolegend |  |
| F4/80 | APC | BM8 | 17-4801-82 | eBioscience |  |
| CD122 | PerCP-eFluor710 | TM-b1 | 46-1222-82 | eBioscience |  |
| Gr1 | FITC | RB6-8C5 | 108406 | Biolegend |  |
| TIGIT | PerCP-eFluor710 | GIGD7 | 46-9501-82 | eBioscience |  |
| PD1 | APC Cy7 | 29F.1A12 | 135224 | Biolegend |  |
| Lag3 | PE | C9B7W | 12-2231-83 | eBioscience |  |
| OX40 | PE Cy7 | OX86 | 119416 | Biolegend |  |
| 41BB | FITC | 1AH12 | 558975 | BD Biosciences |  |
| GITR | BV421 | 108-17 | 371208 | Biolegend |  |
| CD44 | BV421 | IM7 | 563970 | BD Biosciences |  |
| CD69 | APC | H1.2F3 | 104514 | Biolegend |  |
| CD25 | APC Cy7 | PC61 | 102026 | Biolegend |  |
| CD103 | PerCP-eFluor710 | 2E7 | 46-1031-82 | eBioscience |  |
| PDL1 | PE | 10F.9G2 | 124308 | Biolegend |  |
| I-A/I-E | PE Cy7 | M5/114.15.2 | 107630 | Biolegend |  |
| CD80 | BUV496 | 16-10A1 | 741091 | BD Biosciences |  |
| anti-IL-36R (anti-IL1RL2) | Unconjugated | Polyclonal | 38013 | Invitrogen | IL-36R expression |
| anti-IL-36R (anti-IL1RL2) | Unconjugated | Polyclonal | 7501 | ProSci |  |
| Anti-NK1.1 | Unconjugated | PK136 | BE0036 | BioXCell | In-vivo NK depletion |
| Mouse IgG2a isotype | Unconjugated | C1.18.4 | BE0085 | BioXCell | Isotype control |
| Anti-Ly6G | Unconjugated | 1A8 | BE0075-1 | BioXCell | In-vivo neutrophil depletion |
| rat IgG2a isotype | Unconjugated | 2A3 | BE0089 | BioXCell | Isotype control |
| Anti-Gr1 | Unconjugated | RB6-8C5 | BE0075 | BioXCell | In-vivo neutrophil and monocyte depletion |
| rat IgG2b isotype | Unconjugated | LTF-2 | BE0090 | BioXCell | Isotype control |
| Ly6G | PE | 1A8 | 127607 | Biolegend | Sort live neutrophils |
| Sytox Blue | Sytox Blue | N/A | S34857 | Thermo Fisher Scientific | Sort live cells |
| CD11c | FITC | N418 | 117305 | Biolegend | Sort DC1, DC2 cells |
| MHC Class II | PECy7 | M5/114.15.2 | 107629 | Biolegend |  |
| XCR1 | APC | ZET | 148205 | Biolegend | Sort DC1 cells |
| CD172a (Sirpa) | PerCP-eFluor710 | P84 | 46-1721-82 | eBioscience | Sort DC2 cells |
| NK1.1 | FITC | PKI36 | 108705 | Biolegend | Sort NK cells |
| Phospho-p38 MAPK | N/A | D3F9 | 4511 | Cell Signaling Technology | Primary (Western Blot) |
| p38 MAPK | N/A | D13E1 | 8690 | Cell Signaling Technology |  |
| Phospho-IκBα | N/A | 5A5 | 9246 | Cell Signaling Technology |  |
| IκBα | N/A | L35A5 | 4814 | Cell Signaling Technology |  |
| Phospho-NF-κB p65 | N/A | 93H1 | 3033 | Cell Signaling Technology |  |
| NF-κB p65 | N/A | D14E12 | 82425 | Cell Signaling Technology |  |
| HSP90 | N/A | C45G5 | 4877 | Cell Signaling Technology |  |
| Anti-rabbit IgG | HRP | N/A | 7074 | Cell Signaling Technology | Secondary (Western Blot) |
| Anti-mouse IgG | HRP | N/A | 7076 | Cell Signaling Technology |  |
| Anti-Armenian Hamster IgG | HRP | N/A | 127-035-160 | Jackson Immunoresearch |  |
